# Hypoxia exposure impairs male fertility via inhibiting Septin2-mediated spermatogonial proliferation

**DOI:** 10.1093/hropen/hoaf027

**Published:** 2025-05-14

**Authors:** Zhibin Li, Shuying Li, Yufeng Xiao, Junfeng Guo, Jianchun Zhou, Yang Chen, Juan Yang, Chunli Gong, Bing He, Yuyun Wu, Nannan Gao, Huan Yang, Limin Gao, Hua Hu, Yunfang Zhang, Shiming Yang

**Affiliations:** Department of Gastroenterology, Xinqiao Hospital, Army Medical University, Chongqing, China; Department of Obstetrics and Gynecology, Xinqiao Hospital, Army Medical University, Chongqing, China; Department of Obstetrics and Gynecology, Xinqiao Hospital, Army Medical University, Chongqing, China; Department of Gastroenterology, Xinqiao Hospital, Army Medical University, Chongqing, China; Department of Gastroenterology, Xinqiao Hospital, Army Medical University, Chongqing, China; Department of Gastroenterology, Xinqiao Hospital, Army Medical University, Chongqing, China; Key Laboratory of Biorheological Science and Technology, Ministry of Education, College of Bioengineering, Chongqing University, Chongqing, China; Department of Obstetrics and Gynecology, Xinqiao Hospital, Army Medical University, Chongqing, China; Department of Gastroenterology, Xinqiao Hospital, Army Medical University, Chongqing, China; Department of Gastroenterology, Xinqiao Hospital, Army Medical University, Chongqing, China; Department of Gastroenterology, Xinqiao Hospital, Army Medical University, Chongqing, China; Department of Gastroenterology, Xinqiao Hospital, Army Medical University, Chongqing, China; Department of Gastroenterology, Xinqiao Hospital, Army Medical University, Chongqing, China; Department of Gastroenterology, Xinqiao Hospital, Army Medical University, Chongqing, China; Department of Obstetrics and Gynecology, Xinqiao Hospital, Army Medical University, Chongqing, China; Clinical and Translational Research Center of Shanghai First Maternity and Infant Hospital, Shanghai Key Laboratory of Signaling and Disease Research, Frontier Science Center for Stem Cell Research, School of Life Sciences and Technology, Tongji University, Shanghai, China; Sycamore Research Institute of Life Sciences, Shanghai, China; Department of Gastroenterology, Xinqiao Hospital, Army Medical University, Chongqing, China

**Keywords:** hypoxia, spermatogonia, proliferation, Septin2, PP2A /AKT

## Abstract

**STUDY QUESTION:**

What are the molecular mechanisms underlying hypoxia-induced male reproductive impairment?

**SUMMARY ANSWER:**

Hypoxia compromises Septin2 (*Sept2*) transcription in spermatogonia, which impedes spermatogonial proliferation through protein phosphatase 2A (PP2A)-dependent AKT dephosphorylation.

**WHAT IS KNOWN ALREADY:**

Hypoxia is associated with impaired spermatogenesis and poor sperm parameters in men. Spermatogonia proliferation, a crucial early step in spermatogenesis, is essential for maintaining the spermatogenic cell population and ensuring sperm quality. However, the connection between hypoxia and spermatogonial proliferation remains poorly understood, and treatment options for hypoxia-related reproductive disorders are limited.

**STUDY DESIGN, SIZE, DURATION:**

A cross-sectional study analyzed semen samples from 24 high-altitude (HA) residents, 6 pathological hypoxia (PH) patients, and 19 healthy controls to evaluate hypoxia-associated sperm parameter alterations. Complementary animal studies employing a hypobaric chamber-induced hypoxic mouse model (n = 5) confirmed reproductive impairments through assessment of birth rates, sperm quality, and testicular histopathology. Transcriptomic profiling of hypoxic versus normoxic mouse testes (n = 3/group) identified spermatogonial proliferation defects as a predominant pathological feature and pinpointed *Sept2* as a candidate mediator. Subsequent mechanistic investigations employed *in vitro* hypoxic culture of spermatogonial cell lines under hypoxic conditions coupled with pharmacological modulation of PP2A activity in mice (n = 3–5 per intervention group) to delineate the underlying molecular pathways.

**PARTICIPANTS/MATERIALS, SETTING, METHODS:**

Semen parameters were evaluated using computer-assisted sperm analysis (CASA; for sperm concentration, count, and motility), morphological staining (Pap staining for sperm deformity), and eosin–nigrosin staining (for sperm viability). In the hypoxic mouse model, fertility outcomes were assessed through fertility assessment (mating experiments), sperm parameters (CASA), testicular histology (H&E staining), and spermatogonia proliferation (immunohistochemistry and qPCR). In hypoxic spermatogonial cell models, cell proliferation was detected using CCK-8, EdU incorporation, flow cytometry, and western blotting. *Sept2* manipulation (knockdown/overexpression), followed by mechanistic analyses (dual-luciferase reporter assay, DNA pulldown/mass spectrometry, TMT-based quantitative proteomics, co-immunoprecipitation, etc.), was performed to investigate the mechanism underlying hypoxia-regulated spermatogonia proliferation. The SEPT2 inhibitor forchlorfenuron (FCF), the PP2A agonists celastrol, erlotinib, and FTY720, as well as PP2A inhibitor okadaic acid (OA) were used to investigate the role of the SEPT2–PP2A–AKT axis in male fertility regulation.

**MAIN RESULTS AND THE ROLE OF CHANCE:**

Both human populations (HA residents and PH patients) and mouse model consistently demonstrated hypoxia-related reproductive dysfunction. Mechanistic analyses revealed that hypoxia significantly downregulated *Sept2* expression in spermatogonia, concomitant with impaired proliferative capacity. *Sept2* knockdown in normoxic mice phenocopied the hypoxia-induced defects in spermatogenesis. Complementary *in vitro* studies confirmed that *Sept2* depletion impaired spermatogonial proliferation by inducing G1–S phase arrest, while its overexpression mitigated hypoxia-related proliferative defects. Further investigation revealed that hypoxia disrupts *Sept2* transcription by interfering with the binding of RNA polymerase II subunit A (POLR2A) to the *Sept2* promoter. The consequent reduction in *Sept2* expression led to stabilization of the B56γ regulatory subunit of PP2A, resulting in enhanced AKT dephosphorylation and subsequent suppressed spermatogonial proliferation. Pharmacological intervention with the PP2A inhibitor OA restored reproductive competence and sperm quality in hypoxic mice, whereas PP2A agonists exacerbated these deficits.

**LARGE SCALE DATA:**

RNA-seq data are deposited in China National Center for Bioinformation (CNCB) under accession number PRJCA035733.

**LIMITATIONS, REASONS FOR CAUTION:**

This study focused on the effects of hypoxia on sperm parameters. Additional factors such as alterations in reproductive hormones and sexual function may contribute to hypoxia-induced infertility and warrant further research.

**WIDER IMPLICATIONS OF THE FINDINGS:**

This study identifies the SEPT2–PP2A/B56γ–AKT axis as a key regulator in hypoxia-related spermatogonia proliferation impairment. PP2A inhibitors such as OA may offer a therapeutic strategy to protect male fertility under hypoxic conditions.

**STUDY FUNDING/COMPETING INTEREST(S):**

This work was supported by the National Natural Science Foundation of China (No. 82101688) and Natural Science Foundation of Chongqing (No. CSTB2022NSCQ-MSX0943). The authors have no conflicts of interest to declare.

WHAT DOES THIS MEAN FOR PATIENTS? Men exposed to high-altitude regions (where oxygen is thin) or those suffering from health issues that limit oxygen—such as heart problems, lung disease, or anemia—often struggle with poorer sperm quality and fertility challenges. Our research shows that low oxygen disrupts a natural process in cells responsible for sperm production. It can be imagined as a chain reaction: a critical ‘signal’ (called SEPTIN2) weakens, which triggers a ‘brake’ (protein phosphatase 2A) that slows sperm production. As a result, sperm become scarcer and weaker. However, our subsequent experiments in mice showed that a drug called okadaic acid (which helps to lift this ‘brake’) improved sperm health and fertility. On the other hand, other drugs that strengthen the ‘brake’ (such as celastrol, erlotinib, and FTY720) worsened fertility problems, especially in low-oxygen conditions. This research points to a promising new approach for treating low oxygen-related infertility. It also serves as an important warning: some medications may need to be avoided by men trying to conceive, particularly those already dealing with oxygen-related health concerns.

## Introduction

Infertility is becoming increasingly prevalent worldwide, representing a growing public health challenge. Globally, infertility affects an estimated 15% of couples, with male factors accounting for half of all cases (Minhas *et al.*, 2021). Male infertility now represents not merely a clinical condition but a multifaceted societal challenge with profound psychological, social, and economic consequences for individuals and healthcare infrastructures (Agarwal *et al.*, 2021). The causes of male infertility are multifactorial, involving congenital, acquired, and idiopathic factors leading to spermatogenic dysfunction, including anorchia, varicocele, and obesity, among others (Agarwal *et al.*, 2021).

A major contributor to male infertility is a decline in sperm quality, primarily influenced by spermatogenesis, a complex process that occurs within the seminiferous tubules of the testes. Spermatogenesis initiates with mitotic expansion in spermatogonia, followed by meiotic progression in spermatocytes and morphological transformations in spermatids (Rabbani *et al.*, 2022), spanning ∼35 days in mice and 74 days in humans (Adler, 1996). The unique anatomical and physiological features of the seminiferous tubules—specifically, the absence of a direct blood supply and reliance on oxygen diffusion, combined with the high oxygen demand of germ cells—make spermatogenesis exceptionally sensitive to oxygen availability (Lord and Nixon, 2020; Lord, 2025). Hypoxia, defined as a state of oxygen deprivation below physiological levels, can arise from either environmental or pathological origins (Li *et al.*, 2021). Environmental hypoxia predominantly occurs at high altitudes (HAs; >2500 m), where decreased atmospheric oxygen pressure (pO_2_ <60 mmHg) leads to compromised systemic oxygen saturation (SaO_2_ <90%) and subsequent testicular hypoxia (Richalet *et al.*, 2024). Pathological hypoxia (PH) develops through two primary mechanisms: (i) impaired oxygen supply, including pulmonary/respiratory disorders (Nathan *et al.*, 2019) and obstructive sleep apnea (Torres *et al.*, 2014); and (ii) reduced oxygen delivery, including cardiovascular disease (Yu *et al.*, 2022), ischemia (Minas *et al.*, 2023), and varicocele (Gat *et al.*, 2005).

Recent trends indicate an increasing prevalence of hypoxia-related issues in young men. The aggravated burden of hypoxia is attributed not only to the rise in HA workers (Gatterer *et al.*, 2024), but also to the surge in cardiovascular diseases, pulmonary disorders, and respiratory ailments—exacerbated by factors such as the coronavirus disease (COVID-19) pandemic, obesity, unhealthy lifestyles, and environmental pollution (Ortega *et al.*, 2016; Shah *et al.*, 2022; Munzel *et al.*, 2023). Studies in both humans and animals have demonstrated decreased sperm quality following exposure to HAs or PH (Li *et al.*, 2021). However, the underlying mechanisms remain poorly understood, impeding the development of effective therapeutic interventions.

This study aimed to investigate the mechanisms through which hypoxia affects sperm parameters. Semen samples were collected from men exposed to HAs and PH to evaluate alterations in sperm quality. A hypoxic mouse and cell model was developed to explore the underlying mechanisms. Our findings revealed impaired spermatogonial proliferation accompanied by expression of Septin2 (*Sept2*), a gene crucial for cell cycle regulation (Kremer *et al.*, 2007), under hypoxic conditions. We demonstrated that *Sept2* is essential for spermatogonial proliferation and sperm quality. Further analysis revealed how hypoxia suppresses *Sept2* expression and elucidated its role in regulating spermatogonial proliferation. These results not only provide mechanistic insights into hypoxia-induced male infertility but also highlight potential therapeutic targets to improve fertility in individuals affected by hypoxia.

## Materials and methods

### Human subjects

All experimental procedures were conducted in compliance with the World Medical Association’s Declaration of Helsinki and were approved by the Institutional Ethics Committee of the Second Affiliated Hospital of Army Medical University (Ethical Approval No: 2023YD10701). Study participants were recruited according to the following inclusion criteria: age range of 20–40 years; body mass index (BMI) ranging from 18 to 25 kg/m^2^; no history of smoking, alcohol abuse, radiation, or harmful substance exposure within the past year; no reproductive system infection; no family history of genetic disorders; with normal testicular development. All HA participants were originally from plains and had been exposed to hypoxia at an altitude of 4000 m (Tibetan Plateau, China) for over 6 months. For the normoxic healthy control group, only individuals with documented normal fertility history (either through natural conception or as male partners in IVF cycles where female-factor infertility was confirmed and male-factor was excluded) and normal sperm parameters were included. These parameters were defined as follows: ejaculated volume ≥1.5 ml, sperm concentration ≥15 million/ml, total sperm count ≥39 million/ejaculate, progressive motility (PR) ≥32%, total motility (PR plus non-PR, PR + NP) ≥40%, normal morphology ≥4%, and vitality ≥58%. The clinical characteristics of the study participants are provided in Supplementary Table S1.

### Semen sample collection and analysis

#### Semen collection

Semen samples were collected from participants exposed to HA hypoxia (n = 24, Tibetan Plateau, China), PH (n = 6, Chongqing, China), and from healthy control volunteers (n = 19, Chongqing, China) at the Andrology Laboratory. Following a 2- to 7-day abstinence period, semen specimens were collected by masturbation and immediately transported to the laboratory under temperature-controlled conditions (37°C). All analyses were performed within 60 min after collection to minimize pre-analytical variability.

#### Conventional sperm parameter determination

Semen analysis was conducted in strict accordance with the World Health Organization (WHO) Laboratory Manual for the Examination and Processing of Human Semen (5th edition) standards. Following 30 min of liquefaction at 37°C, the sample volume was measured precisely using sterile serological pipettes. Comprehensive sperm evaluation was performed using computer-assisted sperm analysis (CASA; SCA-H-01P system, Microptic, Barcelona, Spain), including the following parameters: sperm concentration ( × 10^6^/ml), total sperm count ( × 10^6^/ejaculate), and motility parameters (PR and PR + NP). All assessments were carried out using standardized microcell slides following the manufacturer’s operational protocols. A minimum of 200 sperm were analyzed per sample, with total analysis time for each sample lasting ∼5 min.

#### Sperm morphology

Morphological assessment followed strict WHO guidelines. Methanol-fixed smears were Pap-stained (G2571, Solarbio, Beijing, China) and independently evaluated by two andrologists using standardized criteria. Inter-observer variability was controlled through duplicate assessments with a variance tolerance of <10%. A minimum of 200 spermatozoa per sample were evaluated using an optical microscope (Olympus, Tokyo, Japan) at  × 400 magnification. Morphological abnormalities were classified as head deformity and tail deformity.

#### Sperm viability

Sperm viability was assessed using eosin–nigrosin staining. Liquefied semen was gently mixed with an equal volume of eosin–nigrosin staining solution for 30 s, smeared onto a slide, air-dried, and examined under a microscope (Olympus) at  × 1000 magnification. Spermatozoa with uncolored heads were considered viable, whereas those with pale pink heads were considered non-viable. A minimum of 200 sperm per sample were evaluated.

### Animals and treatments

#### Mice

Male C57BL/6 mice (6–8 weeks old) were obtained from the specific pathogen-free (SPF) facility at the Army Medical University Center for Laboratory Animals. The animals were maintained under controlled environmental conditions: a constant temperature of 22–24°C; a relative humidity of 50–70%; a 12-h light/dark cycle; free access to sterilized drinking water and standard laboratory chow. This study was performed following the ethical standards of Army Medical University (Approval No. AMUWEC20210124) and in compliance with national laboratory animal welfare regulations.

#### Hypoxic mouse model

Male mice (6–8 weeks old) were housed in a hypobaric oxygen chamber (∼11.2% O_2_) for 35 consecutive days, while age-matched control mice were housed under normoxic conditions (∼21% O_2_) in SPF facilities (n = 5 per group, with three repetitions). The effectiveness of the hypoxia model was evaluated by analyzing *Vegf* and *Glut1* expression levels in the testes, and measuring the right ventricular hypertrophy index (RVHI), calculated as RVHI = [(RV weight)/(LV + septum weight)] × 100%. RV, LV, and Septum represent right ventricle, left ventricle, and interventricular septum, respectively.

#### Sept2 inhibitor forchlorfenuron treatment

Male mice (6–8 weeks old) were administered the *Sept2* inhibitor forchlorfenuron (FCF) (68157-60-8, Aladdin, Shanghai, China) via daily intraperitoneal injection (50 mg/kg/day) for 35 consecutive days under SPF conditions. Age-matched control mice received intraperitoneal injections of an equal volume of the vehicle under identical conditions.

#### Protein phosphatase 2A inhibitor okadaic acid treatment

Male mice (6–8 weeks old) subjected to hypoxia were concurrently treated with intraperitoneal injections of okadaic acid (OA) (S1786, Beyotime, Shanghai, China; 50 μg/kg/day) for 35 days. Normoxic age-matched mice received an equal volume of vehicle as negative controls.

#### Protein phosphatase 2A agonists celastrol, erlotinib, and FTY720 treatments

Male normoxic or hypoxic mice (6–8 weeks old) were concurrently administered intraperitoneal injections of celastrol (HY-13067, MedChemExpress, Monmouth Junction, NJ, USA; 2 mg/kg/day), erlotinib (HY-50896, MedChemExpress; 2 mg/kg/day), or FTY720 (HY-11063, MedChemExpress; 2 mg/kg/day) for 35 days. Age-matched normoxic control mice received an equal volume of vehicle as negative controls.

All agents (FCF, OA, celastrol, erlotinib, and FTY720) were initially dissolved in dimethyl sulfoxide (DMSO) and subsequently diluted in vehicle solution (5% DMSO, 40% PEG300, 5% Tween 80, and 50% normal saline) to achieve working concentrations. Each mouse received a 200-μl injection volume containing the specified drug dosage. Supplementary Table S2 provides comprehensive details of all reagents used in this study.

### Fertility assessment

Male mice were paired with sexually mature female mice in a 1:2 ratio under normoxic SPF conditions for 7 days. Then, the mice were separated until the first litter of pups was born. Fertility was evaluated based on live birth rate. The number and sex ratio of pups in the first litter were recorded for each female.

### Sperm collection and parameters evaluation

#### Sperm collection

Male mice were euthanized and the cauda epididymides were excised and placed in centrifuge tubes containing 1 ml of preheated (37°C) human tubal fluid medium (MR-070-D, Merck Millipore, Billerica, MA, USA). The cauda epididymides were immediately minced with scissors and then incubated for 5 min at 37°C with inversion-mixing several times per minute to release sperm.

#### Sperm count and motility analysis

A 10-μl aliquot of well-mixed sperm suspension was carefully pipetted into a pre-warmed (37°C) counting chamber (SA-4, SUIPLUS, Beijing, China) and analyzed using a CASA system (SSA-Ⅱ PLUS, SUIPLUS, Beijing, China). At least 200 sperm were evaluated per mouse.

#### Sperm morphology assessment

Sperm samples were prepared by smearing 10 μl aliquots of semen onto glass slides, followed by fixation with absolute methanol for 30 min and staining with 2% eosin solution for 1–2 h. Sperm observation and imaging were performed using a microscope (Olympus). Sperm head abnormalities were categorized as blunt hooks, two heads, or round heads, and sperm tail abnormalities were categorized as two tails, folded tails, or short tails. Two independent researchers performed blinded assessments of the same smear, and their results were averaged to determine the final abnormality rates.

#### Sperm viability assessment

A 50-μl aliquot of freshly prepared sperm suspension was carefully mixed with an equal volume of eosin–nigrosin staining solution by gentle pipetting for precisely 30 s, after which 10 μl of the mixture was uniformly smeared onto a pre-cleaned microscope slide using the wedge technique, allowed to air-dry completely for 10 min at room temperature (25 ± 1°C), and subsequently examined under oil immersion at  × 1000 magnification using a microscope (Olympus). Spermatozoa with unstained heads were considered live, whereas those with pale pink heads were classified as dead. At least 200 sperm were analyzed per sample.

### Histological and morphometric analysis of testes

The testes were rinsed in cold phosphate-buffered saline (PBS) and fixed overnight in Bouin’s solution. After fixation, the testes were embedded in paraffin and sectioned into 5-μm-thick histological slices. The largest cross-section of each testis was selected for histological and morphometric analyses.

#### Immunofluorescence analysis

Tissue sections were processed through the following sequential steps: (i) deparaffinization in xylene (3 changes, 5 min each), (ii) rehydration through a graded ethanol series (100%, 95%, 80%, 70%; 2 min each), and (iii) antigen retrieval in 10 mM sodium citrate buffer (pH 6.0) at 95°C for 20 min. After cooling to room temperature, sections were permeabilized with 0.1% Triton X-100 in PBS for 10 min at 4°C. Non-specific binding was blocked with 5% normal goat serum in PBS for 1 h at room temperature before overnight incubation at 4°C with primary antibodies diluted in blocking solution. Nuclei were counterstained with DAPI (1 μg/ml) for 5 min.

#### Immunohistochemical staining

Antigen retrieval and membrane permeabilization were performed as described above. Sections were blocked with a solution containing 3% BSA and 10% donkey serum in PBS. Primary antibody incubation was performed overnight at 4°C, followed by secondary antibody incubation for 1 h at room temperature. Primary antibodies used for immunofluorescence (IF) and immunohistochemistry (IHC) are listed in Supplementary Table S3.

#### Morphometric analysis

Dewaxed sections underwent routine hematoxylin staining for 5 min, followed by eosin counterstaining for 1 min. Subsequent dehydration and mounting steps were performed prior to microscopic examination.

Images were captured using a microscope (Olympus). For each testicular sample, two sections were analyzed, and only cross-sections of seminiferous tubules with an aspect ratio (length-to-width ratio) of less than 1.2 were included in the statistical analysis. The results are presented as the average of all eligible seminiferous tubules from the two sections per testis.

### Cell culture and treatments

#### Cell culture

The mouse spermatogenic cell lines GC-1 (spermatogonia-derived, GC-1 spg) and GC-2 (spermatocyte-derived, GC-2 spd) were acquired from the American Type Culture Collection (ATCC). These cells were maintained in Dulbecco’s Modified Eagle Medium (SH30022.01, HyClone, Logan, UT, USA) containing 10% fetal bovine serum (A5256701, Gibco, Grand Island, NY, USA) and antibiotic solution (100 IU/ml penicillin, 100 μg/ml streptomycin, and 50 μg/ml gentamicin). Cell cultures were incubated at 37°C under saturated humid conditions.

#### Hypoxia treatment

GC-1 and GC-2 cells (1 × 10^6^ cells/well) were plated in 6-well culture plates and allowed to adhere for 12 h under normoxic culture conditions (21% O_2_, 5% CO_2_, 37°C) in a CO_2_ incubator (Thermo Fisher Scientific, Waltham, MA, USA). Following attachment, cells underwent three gentle washes with warm PBS (37°C), followed by fresh medium replacement. The cultures were then transferred to a tri-gas incubator (Thermo Fisher Scientific) set to maintain hypoxic conditions (1% O_2_, 5% CO_2_, balanced with N_2_) for 24 h of continuous exposure.

#### Cell treatments

A total of 10^6^ cells were seeded into 6-well plates and pre-incubated in a normoxic incubator for 12 h. Treatments included the hypoxia-inducible factors (HIF)-1α activator CoCl_2_ (449776, Sigma-Aldrich, St Louis, MO, USA; 200 μM), dimethyloxalylglycine (DMOG) (S7483, Selleckchem, Houston, TX, USA; 100 μM), or the POLR2A inhibitor α-Amanitin (α-Ama) (MED11734, Medbio, Shanghai, China; 1 μg/ml) for 24 h. SC79 (SF2730, Beyotime, Shanghai, China; 10 μM) or OA (S1786, Beyotime; 20 μM) were added concurrently with hypoxia treatment for 24 h. To investigate the role of *Sept2* in regulating protein phosphatase 2A (PP2A)/B56γ degradation, the proteasome inhibitor MG132 (133407-82-6, Aladdin, Shanghai, China; 10 μM) was added for 6 h, and the protein synthesis inhibitor cycloheximide (CHX) (HY-12320, MedChemExpress, Monmouth Junction, NJ, USA; 5 μg/ml) was added in four different configurations: for 0, 3, 6, and 9 h. Cells were harvested immediately after treatment for further experiments. Details of the reagents used in this study are provided in Supplementary Table S2.

### Cell transfections

siRNAs against *Sept2*, *Polr2a*, *Phf8*, *Trim28*, *Max*, *Hdac1*, *Cebpz*, *Ewsr1, and Hif-1α*, along with negative control (nc) siRNA were synthesized by Sangon (Shanghai, China) (siRNA sequences are listed in Supplementary Table S4). The following DNA sequences were synthesized directly by Sangon and subcloned into the PCMV5 backbone: *Sept2*, HA-targeted B56γ, and HA-targeted B56γ-mut (K64A, K183A, K333A, K413A, K432A). The binding sites were predicted by CUCKOO (Wang *et al.*, 2022) (https://biocuckoo.cn/) and PhosphoSitePlus (Hornbeck *et al.*, 2015) (https://www.phosphosite.org/). Lipofectamine 8000 (C0533, Beyotime Biotechnology, Shanghai, China)-mediated transfection was performed at 30–40% confluency using either siRNA (100 pmol) or plasmid DNA (2.5 μg) per well. Complexes were formed in Opti-MEM (Lipofectamine 8000: 4 μl/well) and incubated for 6 h before medium replacement. Cells were analyzed 24–48 h post-transfection.

### RNA extraction, real-time quantitative PCR, and RNA sequencing

Total RNA was isolated from fresh testicular tissue, epididymal samples, or cultured cells using TRIzol reagent (9109; Takara Bio, Shiga, Japan). RNA concentration and purity were determined spectrophotometrically (NanoDrop 2000; Thermo Fisher Scientific). Reverse transcription was performed using 1 μg total RNA with the PrimeScript RT Reagent Kit (RR047A, Takara Bio, Shiga, Japan), and quantitative PCR (qPCR) was conducted using a TB Green Premix Ex Taq (RR420A, Takara Bio). Mous*e β-actin* (*Actb*) and *Gapdh* were used as internal normalization controls. Relative gene expression was calculated using the 2^−ΔΔCt^ method. The primer sequences are presented in Supplementary Table S5.

RNA sequencing libraries were constructed using the TruSeq Stranded mRNA LT Sample Prep Kit (Illumina, San Diego, CA, USA) according to the manufacturer’s instructions. Sequencing was subsequently performed on the Illumina NovaSeq 6000 platform. Raw sequencing data underwent preprocessing with Fastp (Chen, 2023) to trim adapters, filter repetitive sequences, and discard low-quality reads. Differential gene expression profiling was executed using the Cufflinks–Cuffmerge pipeline (Trapnell *et al.*, 2012), applying thresholds of |fold change | >1.5 and adjusted *P*-value <0.05 to identify statistically significant expression changes.

### Tandem mass tag-based quantitative proteomics

Tandem mass tag (TMT)-based quantitative proteomics was employed to profile protein expression in GC-2 cells derived from *Sept2* knockdown and control groups. Cellular lysates were prepared using SDT buffer, and protein concentrations were determined via BCA Protein Assay Kit (Bio-Rad, Hercules, CA, USA). Following filter-aided sample preparation (FASP digestion), 100 μg of peptide mixture per sample was labeled with TMT reagents according to the manufacturer’s protocol (Thermo Fisher Scientific).

Labeled peptides underwent high-pH reversed-phase fractionation using a Reversed-Phase Peptide Fractionation Kit (Thermo Fisher Scientific). Briefly, fractions were reconstituted, acidified with 0.1% TFA, and processed on a high-pH reversed-phase spin column. Hydrophobic peptides were retained on the resin, desalted with water, and eluted using a stepwise acetonitrile gradient in alkaline conditions. Ten collected fractions were subsequently desalted using C18 cartridges (Empore SPE Cartridges C18, Sigma-Aldrich, St Louis, MO, USA) and concentrated via vacuum centrifugation.

LC-MS/MS analysis was conducted on a Q Exactive mass spectrometer (Thermo Fisher Scientific) interfaced with an Easy nLC system (Thermo Fisher Scientific). Peptides were loaded onto a reverse-phase trap column, resolved on a C18 analytical column, and separated using a linear gradient of buffer A (0.1% formic acid) and buffer B (84% acetonitrile, 0.1% formic acid) at 300 nl/min. The instrument was operated in positive ion mode, employing a data-dependent top10 method (Michalski *et al.*, 2011) to prioritize precursor ions for HCD fragmentation.

Raw LC-MS/MS data were processed using the MASCOT engine (Matrix Science, London, UK; version 2.2) (Perkins *et al.*, 1999) integrated into the Proteome Discoverer 1.4 (Thermo Fisher Scientific) (Proteome Discoverer, 2023). Tandem mass spectra were searched against the UniProt database (UniProt Consortium, 2021) (https://www.uniprot.org/) with a false discovery rate threshold of 5% and a minimum score of 40 for modified peptides. Proteins were considered differentially expressed if they exhibited a fold change >1.2 or <0.83.

### Western blotting

Western blotting was performed according to the standard protocols. Briefly, proteins from testis samples or cells were extracted with RIPA buffer (containing 200 μM PMSF and 1 mM phosphatase inhibitor), quantified by BCA Protein Assay Kit (P0009, Beyotime, Shanghai, China), and separated by SDS–PAGE (30 μg/lane). After transfer to PVDF membranes, blots were probed with primary antibodies (Supplementary Table S3) and HRP-secondaries, with detection by ECL reagents (WBKLS0050, Merck Millipore, Billerica, MA, USA).

### Proliferation assays

#### CCK-8 assay

Cells were plated in 96-well culture plates at an initial density of 2 × 10³ cells/well in 100 μl complete medium. After cell attachment (24 h), proliferation was assessed using the Cell Counting Kit-8 (CCK-8; HY-K0301, MedChemExpress). Briefly, 10 μl of CCK-8 reagent was added to each well followed by incubation for 1–2 h at 37°C in a humidified 5% CO_2_ atmosphere. Absorbance was measured at 450 nm using a microplate reader (Thermo Fisher Scientific). Absorbance values were normalized to the baseline cell number (Day 0).

#### EdU incorporation assay

EdU incorporation assays were conducted using the iClick EdU Andy Fluor 594 Imaging Kit (A005, GeneCopoeia, Rockville, MD, USA). Briefly, cells cultured to 50% confluence were pulse-labeled with 10 μM EdU in complete medium for 2 h at 37°C under standard culture conditions. Following labeling, cells were fixed (3.7% formaldehyde, 15 min), permeabilized (0.5% Triton X-100, 20 min), and underwent the click reaction (30 min incubation with Alexa Fluor 594-conjugated detection reagent), and nuclear counterstaining (5 min with 1 μg/ml Hoechst 33342). EdU image was visualized under a fluorescence microscope (Olympus).

### Flow cytometry

Cells cultured in 6-well plates were digested with trypsin and resuspended in 300 μl PBS. Cold anhydrous ethanol (700 μl) was added dropwise to the cell suspension while vortexing, and the mixture was fixed at −20°C for 24–48 h. The fixed cells were washed, and 500 μl of BD Pharmingen PI/RNase Staining Buffer (550825, BD Biosciences, San Jose, CA, USA) was added to the cell pellets. After incubating for 30 min in the dark at 4°C, fluorescence intensity was measured using a Cytoflex S flow cytometer (BD Biosciences), and data analysis was performed using FlowJo™ v10 software (BD Life Sciences) (FlowJo, 2023).

For flow cytometry, forward scatter (FSC) and side scatter (SSC) were used to visualize the cell populations. Setting gate to exclude debris and non-cellular particles based on the FSC and SSC thresholds. PI staining, which intercalates with DNA, was used to assess DNA content. Fluorescence intensity correlated with DNA content: G0/G1-phase cells (2 N DNA) had lower intensity, S-phase cells (2 N-4N DNA) showed intermediate intensity, and G2/M-phase cells (4 N DNA) exhibited higher intensity. Gates were set on the histograms of fluorescence intensity to distinguish G0/G1-phase, S-phase, and G2/M-phase cells. These gates were validated using known cell populations, and cell cycle phase percentages were calculated based on the gated cell counts.

### Luciferase reporter assay

The *Sept2* promoter and a mutant version of the *Sept2* promoter (Δ-1995–1796 bp) were synthesized by Sangon (Shanghai, China) and inserted into the pGL3-Basic vector. GC-2 cells were co-transfected with the luciferase reporter plasmid and the Renilla luciferase reporter plasmid using Lipofectamine 8000. After 24 h, the transfected cells were treated with hypoxia, α-Ama, or si-*Polr2a* for an additional 24 h. Luciferase activity was measured using the Dual-Luciferase Reporter Assay System (E1910, Promega, Madison, WI, USA) according to the manufacturer’s protocol. Firefly luciferase activity was normalized to Renilla luciferase activity, and the results were expressed as the fold change in firefly luciferase activity relative to Renilla luciferase activity.

### DNA pulldown assay and mass spectrometry

Cells cultured in 6-well plates were washed twice with cold PBS and harvested using a cell scraper. Nuclear and cytoplasmic proteins were isolated using a Nuclear and Cytoplasmic Protein Extraction Kit (P0028, Beyotime), according to the manufacturer’s instructions. A 5′ biotin-conjugated full-length *Sept2* promoter, amplified using PCR with PrimeStar Max DNA Polymerase (R022A, Takara Bio) and biotinylated primers, was used as the probe. The non-biotinylated full-length *Sept2* promoter served as a mock control. The PCR products were purified using a PureLink Quick Gel Extraction Kit (K210025, Invitrogen, Carlsbad, CA, USA).

For DNA pulldown, the biotinylated *Sept2* promoter probe was immobilized on streptavidin beads using a Dynabeads Kilobase BINDER Kit (6010, Invitrogen). The immobilized beads were incubated with nuclear or cytoplasmic proteins overnight at 4°C. After washing with cold PBS, the bound proteins were eluted and analyzed by mass spectrometry (MS) or western blotting.

### Co-immunoprecipitation

Cells were washed twice with ice-cold PBS and lysed in NP-40 lysis buffer containing 1 mM PMSF protease inhibitor. After 30 min incubation on ice, lysates were centrifuged at 12 000* g* for 20 min at 4°C to obtain clarified supernatants. Protein concentrations were determined by BCA assay (P0009, Beyotime), and equal amounts (500 μg) of lysates were incubated overnight at 4°C with either anti-SEPT2 or anti-HA antibodies. Immune complexes were captured by incubation with pre-washed protein A/G agarose beads (P5048, Beyotime) for 2–4 h at 4°C. Beads were subsequently washed five times with NP-40 buffer and twice with PBS, followed by protein elution in 2 ×  loading buffer at 95°C for 5 min. Eluted proteins were analyzed by western blotting.

### Chromatin immunoprecipitation

Chromatin immunoprecipitation (ChIP) assays were performed using a Pierce Magnetic ChIP Kit (26156, Thermo Fisher Scientific) following the manufacturer’s instructions. Adherent cells were cross-linked with 1% formaldehyde for 10 min, and cross-linking was quenched with a glycine solution. Cells were harvested, lysed, and subjected to MNase digestion. A 10% aliquot of the digested chromatin lysate was retained as the input sample, and the remaining lysate was immunoprecipitated using an anti-POLR2A antibody. Normal rabbit IgG was used as the negative control. The DNA bound to POLR2A was eluted and purified using IP elution and DNA-binding buffers, respectively.

Purified DNA was quantified using a Nanodrop 2000 spectrophotometer, and the PCR products were analyzed using agarose gel electrophoresis. Primer sequences are listed in Supplementary Table S5.

### Malondialdehyde assay

Malondialdehyde (MDA) levels were quantified using a commercial Lipid Peroxidation MDA Assay Kit (S0131S, Beyotime). Briefly, testis tissues were homogenized in ice-cold RIPA buffer containing 200 μM PMSF using a tissue homogenizer. The homogenates were centrifuged at 12 000 *g* for 20 min at 4°C to obtain clear supernatants. Protein concentrations were determined using a BCA Protein Assay Kit (P0009, Beyotime). For MDA detection, the MDA assay working solution was prepared according to the kit instructions and added to the supernatant. The mixture was heated at 100°C for 15 min in a heating block, cooled on ice for 10 min, and centrifuged at 1000 *g* for 10 min. The absorbance of the supernatant was measured at 532 nm using a microplate reader (Thermo Fisher Scientific). MDA concentrations were calculated using a standard curve and normalized to total protein content (mol MDA/g protein).

### Terminal deoxynucleotidyl transferase dUTP nick end labeling assay

Apoptotic cells were detected using the One Step TUNEL Apoptosis Assay Kit (C1089, Beyotime). Briefly, paraffin-embedded testicular sections (5 μm thickness) were deparaffinized in xylene and rehydrated through a graded ethanol series. After three PBS washes, tissue sections were permeabilized with proteinase K (20 μg/ml in PBS) for 15 min at 37°C. Sections were then incubated with 50 μl terminal deoxynucleotidyl transferase dUTP nick end labeling (TUNEL) reaction mixture (containing terminal deoxynucleotidyl transferase and fluorescein-labeled dUTP) for 60 min at 37°C in a humidified chamber protected from light. Following three additional PBS washes (5 min each), nuclei were counterstained with DAPI (1 μg/ml, 5 min). Fluorescent images were captured using an Olympus BX53 microscope.

### Bioinformatic and statistical analyses

Gene Ontology analysis (Ashburner *et al.*, 2000) was conducted using the October 2022 GO release (http://geneontology.org/), and Kyoto Encyclopedia of Genes and Genomes (KEGG) enrichment analysis (Kanehisa *et al.*, 2012) was performed using KEGG Release 2022/10 (https://www.kegg.jp/). Normality was assessed using Q–Q plots and the Shapiro–Wilk test. Two-tailed Student’s *t*-test was used for comparisons between two groups, and one-way ANOVA was used for comparisons among three or more groups. Sample sizes or repetitions did not exceed to 30. Values are shown as the means ± SDs. Unless otherwise stated, all experiments were repeated three times. Statistical significance is indicated as *P < 0.05, **P < 0.01, ***P < 0.001, ****P < 0.0001; n.s., not significant.

## Results

### Hypoxia impairs sperm parameters in both humans and mice

**Figure 1.**
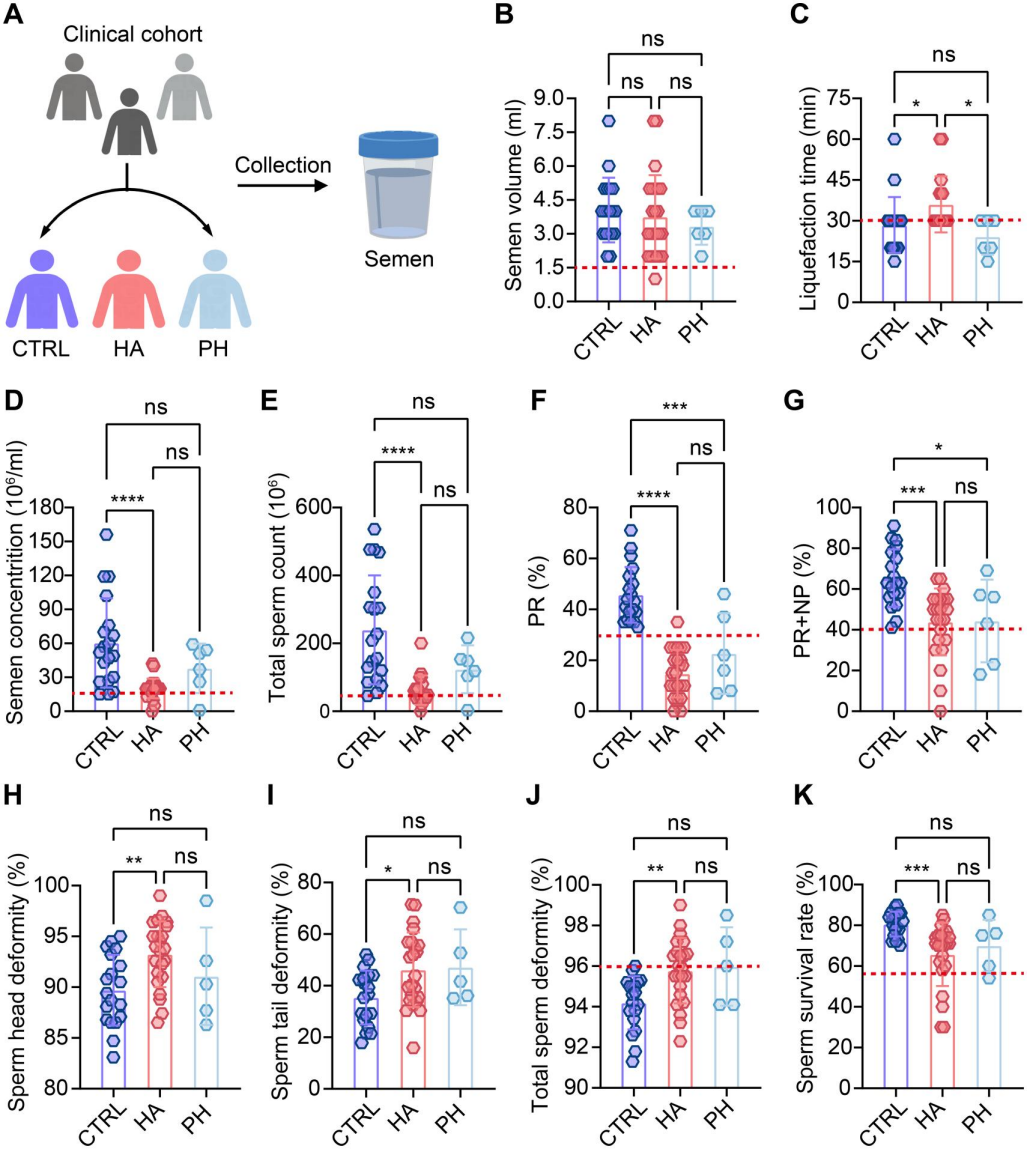
**Changes in semen parameters of participants in high-altitude hypoxia and pathological hypoxia groups**. (**A**) Clinical cohort grouping: CTRL group (n = 19), HA group (n = 24), and PH group (n = 6). (**B**) Semen volumes in men from CTRL, HA, and PH groups. (**C**) Semen liquefaction time in men from the CTRL, HA, and PH groups. (**D–G**) Sperm concentration, total sperm count, PR, and PR + NP in men from the CTRL, HA, and PH groups, as detected by CASA. (**H–J**) Sperm head deformity, sperm tail deformity, and total sperm deformity in men from the CTRL, HA, and PH groups, as detected by pap staining. (**K**) Sperm viability rate in men from the CTRL, HA, and PH groups, as detected by eosin–nigrosin staining. Data were analyzed using one-way ANOVA and presented as mean±SD. *P < 0.05, **P < 0.01, ***P < 0.001, ****P < 0.0001; ns, not significant (*P*>0.05). CTRL, normoxic control; HA, high-altitude hypoxia; PH, pathological hypoxia; PR, progressive motility; NP, non-progressive motility; CASA, computer assisted sperm analysis. The red dashed line represents the WHO sperm criteria for normal (healthy) sperm parameters: ejaculated volume ≥1.5 ml, sperm concentration ≥15 million/ml, total sperm count ≥39 million/ejaculate, PR ≥32%, PR + NP ≥40%, normal morphology ≥4%, and vitality ≥58%.

Semen parameters are key indicators of male reproductive health (Eisenberg *et al.*, 2023). To investigate the effect of hypoxia on semen parameters, we collected semen samples from 24 men living at HA (Tibet Plateau, China), six men with PH (three with anemia, two with apnea syndrome, and one with testicular torsion, Chongqing, China), and 19 healthy controls (CTRL, Chongqing, China) (Fig. 1A). Participant clinical characteristics are presented in Supplementary Table S1. No statistically significant differences in age and BMI were detected across the cohorts (Supplementary Table S1). Semen analysis demonstrated that, despite no significant change in semen volume (4.05 ± 1.43 vs 3.75 ± 1.85, *P* = 0.81), the HA cohort exhibited significant reductions in multiple parameters relative to the CTRL group: sperm concentration (60.37 ± 39.47 vs 20.13 ± 8.70, *P* < 0.0001), total sperm count (239.8 ± 160.5 vs 62.05 ± 43.45, *P* < 0.0001), progressive sperm motility (PR, 45.74 ± 10.92 vs 14.75 ± 9.35, *P* < 0.0001), total sperm motility (PR + NP [non-progressive], 65.32 ± 14.11 vs 43.88 ± 16.43, *P* = 0.0002), and sperm viability rate (80.63 ± 6.07 vs 65.74 ± 15.53, *P* = 0.0009). On the other hand, the HA cohort had a higher semen liquefaction time (28.42 ± 10.28 vs 36.04 ± 10.32, *P* = 0.04), sperm head deformity (89.71 ± 3.49 vs 93.24 ± 3.23, *P* = 0.006), sperm tail deformity (35.41 ± 10.44 vs 46.13 ± 14.47, *P* = 0.029), and total sperm deformity (94.19 ± 1.31 vs 95.77 ± 1.61, *P* = 0.005) (Fig. 1B–K). Similarly, the PH cohort showed reduced PR (45.74 ± 10.92 vs 22.67 ± 15.85, *P* = 0.0001) and total motility (65.32 ± 14.11 vs 44.33 ± 20.27, *P* = 0.02), although other parameters exhibited worsening trends without statistical significance (Fig. 1B–K). Notably, more than 40% of the HA participants (10 out of 24) and over 60% of the PH group (four out of six) were classified as being at high risk for infertility, according to the WHO guidelines (World Health Organization, 2010; Wang *et al.*, 2022), which is significantly higher than the 3–10% incidence rate reported in epidemiological investigations (Eisenberg *et al.*, 2023). Additionally, comparison of semen parameters between the HA and PH cohorts revealed that the HA group exhibited lower values compared to the PH group, although these differences did not reach statistical significance (Fig. 1B–K).

**Figure 2.**
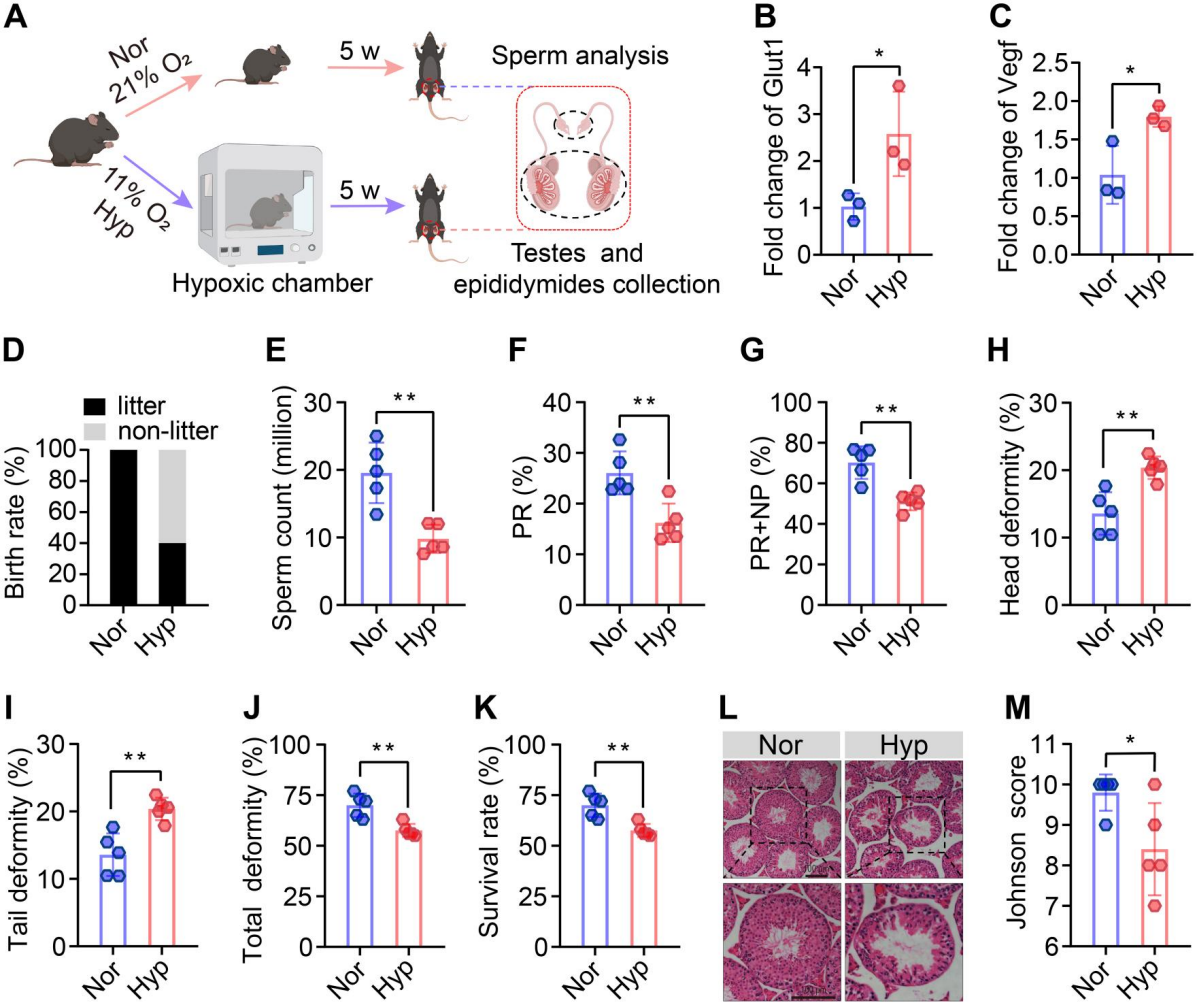
**Reproductive changes in hypoxic mouse model**. (**A**) Male mice aged 6–8 weeks were exposed to a hypoxic chamber for 5 weeks to establish a hypoxic mouse model. (**B, C**) Relative mRNA levels of *Glut1* and *Vegf* in testis from Nor and Hyp mice. (**D**) Fertility assessment showing the birth rate of female mice mated with Nor or Hyp male mice (n = 5 males and 10 females per group). (**E–G**) Sperm count, PR, and PR + NP in Nor or Hyp mice, as detected by CASA (n = 5 per group). (**H–J**) Sperm head deformity, tail deformity, and total sperm deformity in Nor or Hyp mice, as detected by eosin staining (n = 5 per group). (**K**) Sperm viability rate in Nor or Hyp mice, as detected by eosin–nigrosin staining (n = 5). (**L–M**) Representative image of H&E staining of testes from Nor or Hyp mice (n = 5 per group); scale bar = 100 µm. (P) Johnson score based on images of H&E staining (n = 5 per group). Data were analyzed using unpaired Student’s *t*-test and presented as mean±SD. *P < 0.05, **P < 0.01; Nor, normoxia; Hyp, hypoxia; PR, progressive motility; NP, non-progressive motility; d, days, w, weeks, CASA, computer-assisted sperm analysis.

Due to ethical constraints and difficulty in accessing human testes, we established a hypoxic mouse model using a hypobaric chamber (∼11.2% O_2_, vs 21% O_2_ at normoxia) for further study (Fig. 2A). After 5 weeks of hypoxia exposure, the hypoxic mice demonstrated hallmark hypoxia-induced changes, including increased RVHI (Supplementary Fig. S1A), accompanied by elevated mRNA levels of *Glut1* and *Vegf* in the testes (Fig. 2B and C), confirming the effectiveness of the model. We then mated the hypoxic males with naïve (untreated) females at a ratio of 1:2 under normoxic conditions to assess fertility. We found that the birth rate of females mated with hypoxic males was reduced by 60% compared to those mated with control males (Fig. 2D), with no significant differences in number and sex ratio of offspring (Supplementary Fig. S1B and C). Sperm parameter measurements revealed that hypoxia adversely affected sperm count, PR, PR + NP, sperm head morphology, sperm tail morphology, total sperm morphology, and sperm viability rate (Fig. 2E–K). We also observed significant declines in motility parameters, including curvilinear velocity (VCL), straight-line velocity (VSL), average path velocity (VAP), straightness (STR), beat cross frequency (BCF), and amplitude of lateral head displacement (ALH) in hypoxic mice (Supplementary Fig. S1D–I). Histologically, although the testicular index showed no significant changes (Supplementary Fig. S1J), the testes collected from hypoxic mice exhibited architectural changes in seminiferous tubules characterized by thinning of the seminiferous epithelium and partial loss of germ cells (Fig. 2L), accompanied by a reduced Johnson score (Fig. 2M).

The epididymis is a coiled tube located at the back of the testis that plays a critical role in sperm maturation, storage, and transport and provides the proper environment for sperm to develop fertilization capability (James *et al.*, 2020). Next, we performed a transcriptome analysis of epididymides from hypoxic mice to understand the impact of hypoxia on the epididymis. Gene Ontology enrichment analysis of differentially expressed genes (DEGs, fold change >1.5, *P* < 0.05) uncovered that fertility-related processes, including ‘spermatogenesis’, ‘flagellated sperm motility’, ‘fusion of sperm to egg plasma membrane involved in single fertilization’, ‘sperm capacitation’, and ‘binding of sperm to zona pellucida’ were among the top 15, with additional seven (e.g. ‘cilium movement’, ‘microtubule-based movement’, or ‘axonemal dynein complex assembly’) of these 15 processes being key to either structure or function of ciliated cells such as sperm (Supplementary Fig. S1K and L). Histological analysis revealed that hypoxia reduced the thickness of the epididymal epithelium and minimized the number of mature spermatozoa (Supplementary Fig. S1M). Taken together, these unfavorable features of the sperm, testis, and epididymis indicate that hypoxia impedes male reproductive health.

### Hypoxia compromises spermatogonial proliferation

**Figure 3.**
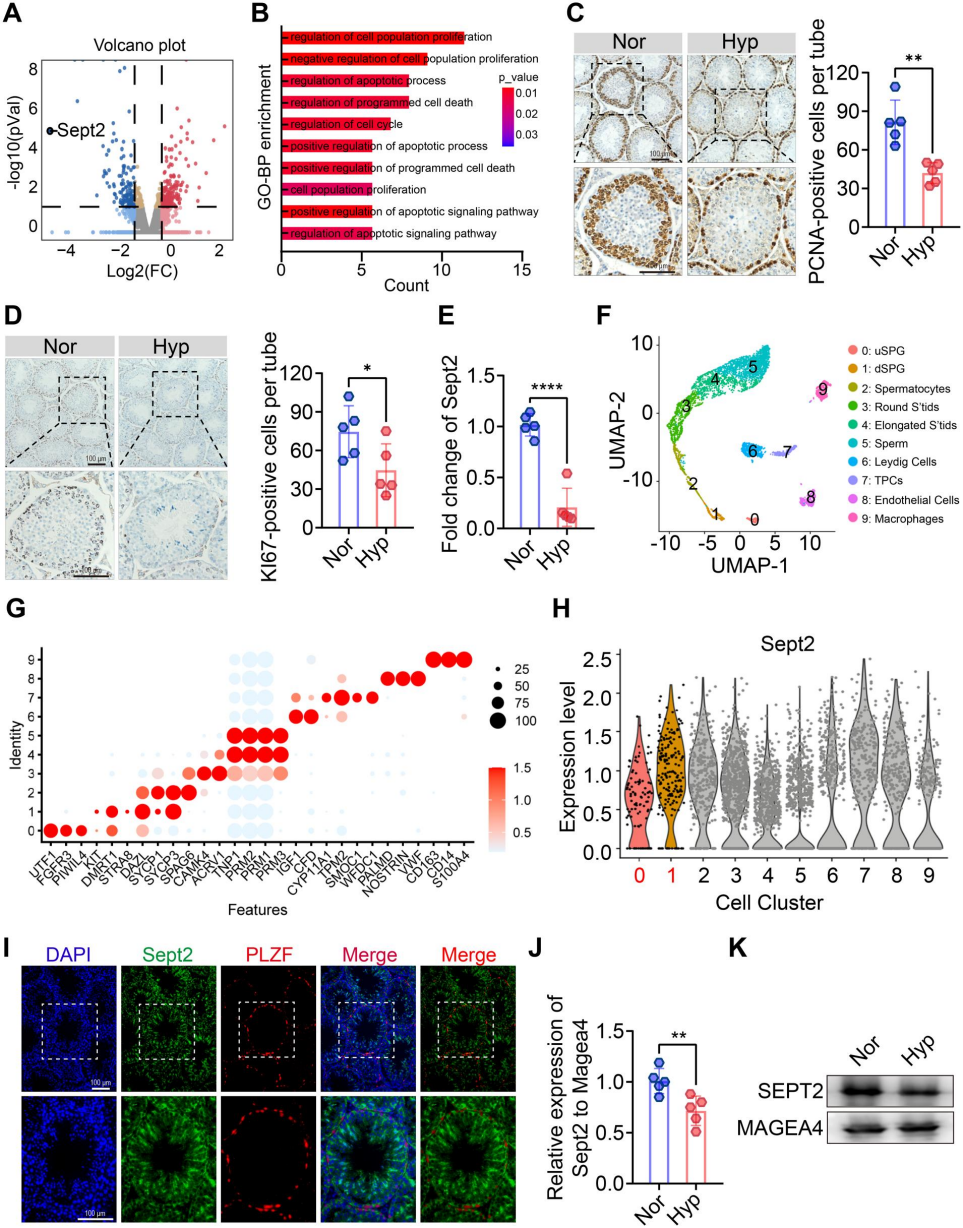
**Hypoxia inhibits spermatogonia proliferation while reducing Septin2 (*Sept2*) expression in spermatogonia**. (**A**) Volcano plot of the differentially expressed genes (DEGs) in testes from Nor and Hyp mice based on RNA-seq. (**B**) Bar plot showing the GO enrichment analysis of the cell proliferation-related DEGs. (**C, D**) Representative immunohistochemical images of PCNA and KI67 in testes from Nor and Hyp mice (n = 5 per group). (**E**) Relative mRNA expression of *Sept2* in testes from Nor and Hyp mice (n = 5 per group). (**F**) Uniform Manifold Approximation and Projection (UMAP) plot of testicular cell types based on single-cell RNA-seq (scRNA-seq). (**G**) Dot plot showing the relative expression of 30 marker genes of different testicular cell types. (**H**) Violin plot showing the expression pattern of *Sept2* mRNA in spermatogonia. (**I**) Representative immunofluorescence images showing the colocalization of SEPT2 with PLZF (promyelocytic leukemia zinc finger protein); scale bar = 100 µm. (**J, K**) Relative mRNA and protein expression of *Sept2* in spermatogonia from Nor and Hyp testes. The data are presented as mean±SD. *P < 0.05, **P < 0.01, ****P < 0.0001. Nor, normoxia; Hyp, hypoxia; FC, fold change; pVal, *P* value; uSPG, undifferentiated spermatogonia; dSPG, differentiating spermatogonia; Round S’tids, round spermids; TPCs, testicular peritubular cells.

To better understand the cellular pathways responsible for hypoxia-induced male fertility impairment, we conducted bulk RNA-seq on testes from normoxic and hypoxic mice (Fig. 3A). GO enrichment analysis of DEGs (*P* < 0.05, fold change >1.5, fragments per kilobase of transcript per million mapped reads >0.1) revealed marked enrichment in cell proliferation-associated processes, including ‘cell cycle’ and ‘cell division’ (Supplementary Fig. S2A). Meanwhile, ‘apoptosis process’ was also among the top enriched processes (Supplementary Fig. S2A). Further detailed GO analysis of DEGs associated with the cell cycle and cell division highlighted that ‘negative regulation of cell population proliferation’ was the second most significantly enriched process (Fig. 3B). Given that spermatogonia are the only spermatogenic cells undergoing mitotic proliferation, we hypothesized that hypoxia negatively regulates spermatogonial proliferation. IHC staining for the proliferation markers PCNA and KI67 showed that testes from hypoxic mice had fewer PCNA- and KI67-positive spermatogonia than those from normoxic mice (Fig. 3C and D). Moreover, the mRNA expression of spermatogonial proliferation-associated genes, including fibroblast growth factor 2 (*Fgf2*), and promyelocytic leukemia zinc finger (*Plzf*), was downregulated in the hypoxic testes (Supplementary Fig. S2B and C). These findings suggest that hypoxia impairs spermatogonial proliferation.

To further validate the adverse effects of hypoxia on spermatogonial proliferation, a well-established hypoxic cell model was utilized with GC-1 cells and GC-2 cells. Proliferation assays showed that hypoxia treatment caused a decrease in cell count and DNA synthesis in GC-1 cells, as shown by the CCK-8 and EdU incorporation assays (Supplementary Fig. S2D and E). As cell proliferation is strictly controlled by cell cycle progression, we detected the cell cycle distribution in hypoxic GC-1 cells. Flow cytometric analysis revealed elevated G0/G1-phase populations with concomitant decreases in S-phase populations (Supplementary Fig. S2F). Hypoxia-induced G1-S phase arrest was further validated by downregulation of core S-phase entry regulators, including cyclin-dependent kinases CDK4 and CDK6, and the key G1 cyclin, cyclin D1 (Supplementary Fig. S2G). Similar phenotypes were also observed in hypoxic GC-2 cells (Supplementary Fig. S2H–K).

Parallel assessment of apoptosis using TUNEL assays, cleaved caspase-3 immunohistochemistry, and western-bloting analysis revealed significantly increased apoptosis in spermatogenic cells of hypoxic mice (Supplementary Fig. S3A–C). Furthermore, elevated MDA levels in hypoxic testicular tissues indicated hypoxia-induced oxidative stress (Supplementary Fig. S3D). These findings implicate apoptosis as another key pathological mechanism contributing to hypoxia-induced male reproductive dysfunction.

### Hypoxia limits spermatogonia proliferation by reducing *Sept2*

To further understand the molecular mechanism by which hypoxia impairs spermatogonial proliferation, we screened the top five genes with the most significant differential expression. *Sept2* emerged as a key candidate due to its high abundance (Supplementary Fig. S4A–C), high conservation (Supplementary Fig. S4D), and the most substantial reduction was observed in hypoxic testes (Fig. 3A). We confirmed by qPCR and immunohistochemistry that *Sept2* levels were downregulated in the hypoxic testes (Fig. 3E and Supplementary Fig. S4E), consistent with the transcriptome profiling data. Notably, the mRNA level of *Sept2* demonstrated a positive relationship with sperm quality and the mRNA expression of *Fgf2* and *Plzf* (Supplementary Fig. S4F), indicating a significant role of *Sept2* in male fertility.

The testis is a heterogeneous tissue with diverse cell types, including spermatogenic, somatic, and immune cells (Wang *et al.*, 2018; Guo *et al.*, 2020; Xia *et al.*, 2024). To determine the expression of *Sept2* in spermatogonia, we analyzed a single-cell RNA sequencing dataset of human testes (GSE112013) (Guo *et al.*, 2018). Using UMAP analysis, we identified 10 distinct testicular cell populations based on specific marker gene expression (Fig. 3F and G). *Sept2* was expressed in both undifferentiated spermatogonia (*UTF1*^+^, *FGFR3*^+^, and *PIWIL4*^+^) and differentiating spermatogonia (*KIT*^+^, *DMRT1*^+^, and *STRA8*^+^) (Fig. 3H and Supplementary Fig. S4G and H). Two-color IF staining of testicular sections further confirmed that SEPT2 colocalized with PLZF, a well-known spermatogonial marker (Fig. 3I). To explore whether hypoxia compromises *Sept2* expression in spermatogonia, we used *Magea4*, a pan-spermatogonia marker (Di Persio *et al.*, 2021), as an internal control for normalization. qPCR and western-blotting analyses demonstrated markedly decreased *Sept2* expression in hypoxic spermatogonia within the testis (Fig. 3J and K).

**Figure 4.**
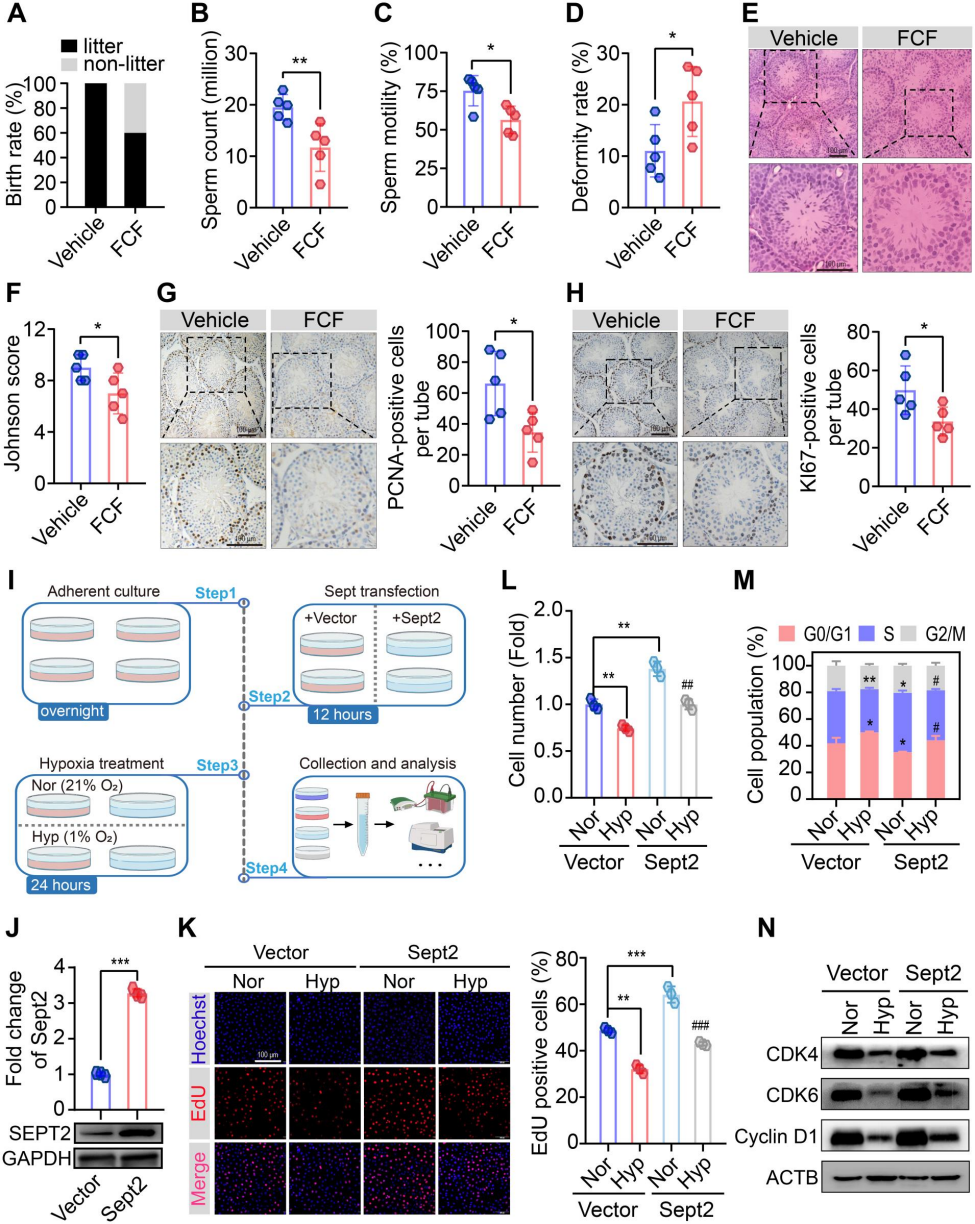
**Septin2 (*Sept2*) regulated spermatogonia proliferation both *in vivo* and *in vitro***. (**A**) Male mice aged 6–8 weeks were treated with FCF or vehicle for 5 weeks. Fertility assessment showing the birth rate of female mice mated with vehicle or FCF male mice (n = 5 males and 10 females per group). (**B, C**) Sperm count and motility in Vehicle and FCF mice, as detected by CASA (n = 5 per group). (**D**) Sperm deformity rate in Vehicle and FCF mice, as detected by eosin staining (n = 5 per group). (**E, F**) Representative image of H&E staining and relative Johnson score of testes from Vehicle and FCF mice; scale bars = 100 µm (n = 5 per group). (**G, H**) Representative immunohistochemical images of PCNA and KI67 in testes from Vehicle and FCF mice; scale bars = 100 µm (n = 5 mice per group). (**I**) Schematic representation of hypoxia treatment and *Sept2* transfection in spermatogonial cell lines. (**J**) The efficiency of *Sept2* overexpression in GC-2 cells. (**K**) representative image of EdU incorporation assay in GC-2 cells. (**L**) Relative cell number of GC-2 cells, as detected by CCK-8; scale bars = 100 µm. (**M**) Cell cycle distribution in GC-2 cells, as detected by flow cytometry. (**N**) Protein levels of CDK4, CDK6, and Cyclin D1 in GC-2 cells. Data were analyzed using unpaired Student’s *t*-test and presented as mean±SD. *P < 0.05, **P < 0.01, ***P < 0.001. ##, ### *P*-values of hypoxia with vector versus hypoxia with *Sept2* overexpression plasmid less than 0.01, and 0.001, respectively. Nor, normoxia; Hyp, hypoxia; FCF, forchlorfenuron; ACTB, β-Actin. CASA, computer-assisted sperm analysis.

Next, we assessed the effects of *Sept2* on spermatogonial proliferation and fertility. Adult male mice were treated with the *Sept2* inhibitor FCF *via* intraperitoneal injections for 35 days under normoxic conditions. Mice treated with FCF exhibited reduced birth rates, impaired sperm parameters, disrupted testicular histology, decreased PCNA- and KI67-positive spermatogonia, and downregulated *Plzf* and *Fgf2* expression (Fig. 4A–H and Supplementary Fig. S5A and B). These findings confirmed that *Sept2* is required for male fertility and spermatogonial proliferation.

*In vitro* experiments with spermatogonial cell lines also demonstrated that hypoxia inhibited *Sept2* mRNA and protein expression in both GC-1 and GC-2 cells (Supplementary Fig. S5C–F). Next, *Sept2* was silenced using siRNA (Supplementary Fig. S5G). The proliferation assay showed that knockdown of *Sept2* reduced cell number (Supplementary Fig. S5H), impaired DNA synthesis (Supplementary Fig. S5I), induced G1-S arrest (Supplementary Fig. S5J), and suppressed the protein expression of CDK4, CDK6, and cyclin D1 in GC-2 cells (Supplementary Fig. S5K). Comparable outcomes were detected in GC-1 cells after *Sept2* knockdown (Supplementary Fig. S6A–E).

We subsequently investigated whether *Sept2* restoration could rescue the hypoxia-induced proliferative deficiency of spermatogonia. After pre-transfection with the *Sept2* overexpression plasmid, GC-2 and GC-1 cells were exposed to hypoxia (Fig. 4I). *Sept2* overexpression increased the cell number, promoted DNA synthesis, restored G1-S progression, and upregulated the protein expression of CDK4, CDK6 and cyclin D1 in both the hypoxic GC-1 (Supplementary Fig. S6F–J) and hypoxic GC-2 cells (Fig. 4J–N). Collectively, our integrated *in vivo* and *in vitro* findings established *Sept2* as a critical regulator of spermatogonial proliferation.

### Hypoxia suppresses *Sept2* transcription in a manner dependent on POLR2A rather than HIF-1α

**Figure 5.**
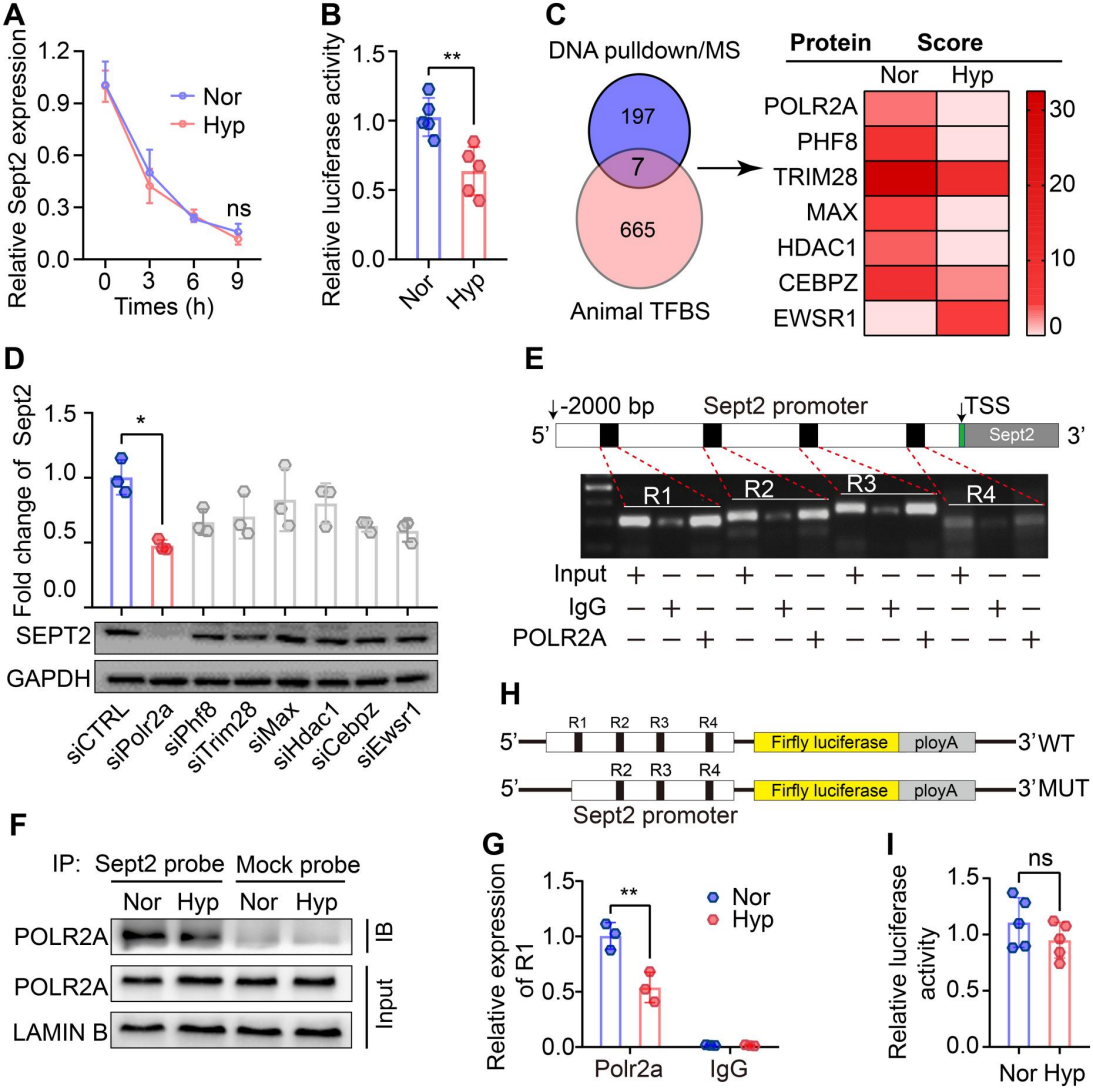
**Hypoxia impairs Septin2 (*Sept2*) transcription by reducing POLR2A binding to *Sept2* promoter**. (**A**) mRNA level of *Sept2* in GC-2 cells with or without hypoxia after treatment with actinomycin D (5 μg/ml) for different time points. (**B**) Dual-luciferase activity of the *Sept2* promoter in Nor and Hyp GC-2 cells. (**C**) Venn diagram showing the overlap of transcription factors identified in the DNA pulldown/MS and AnimalTFDB predictions. (**D**) Relative mRNA and protein expression of *Sept2* in GC-2 cells with various gene knockdowns. (**E**) ChIP-PCR-based agarose gel electrophoresis of different predicted regions. (**F**) DNA pulldown-immunoblotting showing the interaction between POLR2A and the *Sept2* promoter in Nor and Hyp GC-2 cells. (**G**) Relative expression of R1 in GC-2 cells with or without hypoxia treatment. (**H**) Schematic representation of the *Sept2* promoter-mutant plasmid. (**I**) Dual-luciferase activity of *Sept2* promoter-mut plasmid in Nor and Hyp GC-2 cells. Data were analyzed using unpaired Student’s *t*-test and presented as mean±SD. **P < 0.01. Nor, normoxia; Hyp, hypoxia; MS, mass spectrum; ChIP, chromatin immunoprecipitation; R1–R4, region1–region 4; WT, wild-type; MUT, mutant.

We next sought to uncover the mechanism through which hypoxia regulates *Sept2* expression. Given the dramatic reduction in both the mRNA and protein levels of *Sept2* in hypoxic spermatogonia, we hypothesized that hypoxia affects *Sept2* expression at either the transcriptional or the post-transcriptional level. Actinomycin D-based qPCR analysis showed that hypoxia does not influence the stability of *Sept2* mRNA (Fig. 5A). In contrast, a dual-luciferase assay of the *Sept2* promoter revealed that hypoxia significantly reduced *Sept2* transcription (Fig. 5B). HIF-1α serves as a crucial transcription regulator in the hypoxic response, which regulates gene expression through transcriptional activation or inhibition (Gonzalez *et al.*, 2018). We next investigated whether *Sept2* was transcriptionally regulated by HIF-1α. CoCl_2_ and DMOG are two specific HIF-1α activators used to mimic the accumulation of HIF-1α under hypoxic conditions (Regmi *et al.*, 2021; Shi *et al.*, 2021). However, treatment with the HIF-1α-specific activators CoCl_2_ or DMOG had no significant impact on luciferase activity, or the mRNA and protein levels of *Sept2* (Supplementary Fig. S7A–C). Consistently, silencing HIF-1α under hypoxic conditions failed to ameliorate hypoxia-induced downregulation of *Sept2* transcriptional activity, mRNA, and protein expression (Supplementary Fig. S7D–F). These findings suggest that hypoxia suppresses *Sept2* transcription independently of HIF-1α.

To identify the mediators responsible for *Sept2* transcription under hypoxia, we employed a DNA pulldown coupled with MS to identify the transcription factors (TFs) interacting with the *Sept2* promoter (Supplementary Fig. S7G). A total of 204 proteins (*Sept2* promoter probe beads/mock beads >2, relative binding score >1.5) were identified, of which 59 were involved in transcription and 55 participated in transcription regulation (Supplementary Fig. S7H). We then predicted the TF bindings to the *Sept2* promoter using the AnimalTFDB database (Hu *et al.*, 2019) (http://bioinfo.life.hust.edu.cn/AnimalTFDB/). By overlapping the results from the pull-down/MS and data from AnimalTFDB, we identified seven potential TFs for *Sept2* (Fig. 5C). Among these identified potential TFs, only POLR2A silencing (Supplementary Fig. S7I), among the identified potential TFs (Supplementary Fig. S7J), considerably suppressed *Sept2* expression at the transcriptional, mRNA, and protein levels (Supplementary Fig. S7K and Fig. 5D). Similarly, treating GC-2 cells with the *Polr2a* inhibitor α-Ama suppressed *Sept2* transcription and expression (Supplementary Fig. S8A–C). Furthermore, we performed ChIP-PCR based on the four POLR2A binding regions (R1–R4) within the *Sept2* promoter predicted by AnimalTFDB, and confirmed by agarose gel electrophoresis that POLR2A physically interacted with all four regions (Fig. 5E). These results indicate that POLR2A is essential for controlling *Sept2* transcription under hypoxic conditions.

Next, we investigated how hypoxia affected POLR2A expression and found that hypoxia slightly reduced POLR2A protein expression and its nuclear import (Supplementary Fig. S8D and E). A DNA pulldown assay followed by immunoblotting revealed a profound decrease in the interaction between the *Sept2* promoter and POLR2A in hypoxic GC-2 cells (Fig. 5F). These results were further confirmed by ChIP-qPCR analysis, which confirmed that hypoxia impaired the interaction between POLR2A and the region1 (R1) of the *Sept2* promoter (Fig. 5G and Supplementary Fig. S8F–H). Notably, hypoxia did not significantly reduce *Sept2* transcription when R1 was depleted (Fig. 5H and I). Collectively, these results reveal that hypoxia reduced the interaction between POLR2A and the R1 region of the *Sept2* promoter, thereby inhibiting *Sept2* transcription.

### *Sept2* insufficiency suppresses spermatogonia proliferation through enhancing PP2A/B56γ-dependent AKT dephosphorylation

**Figure 6.**
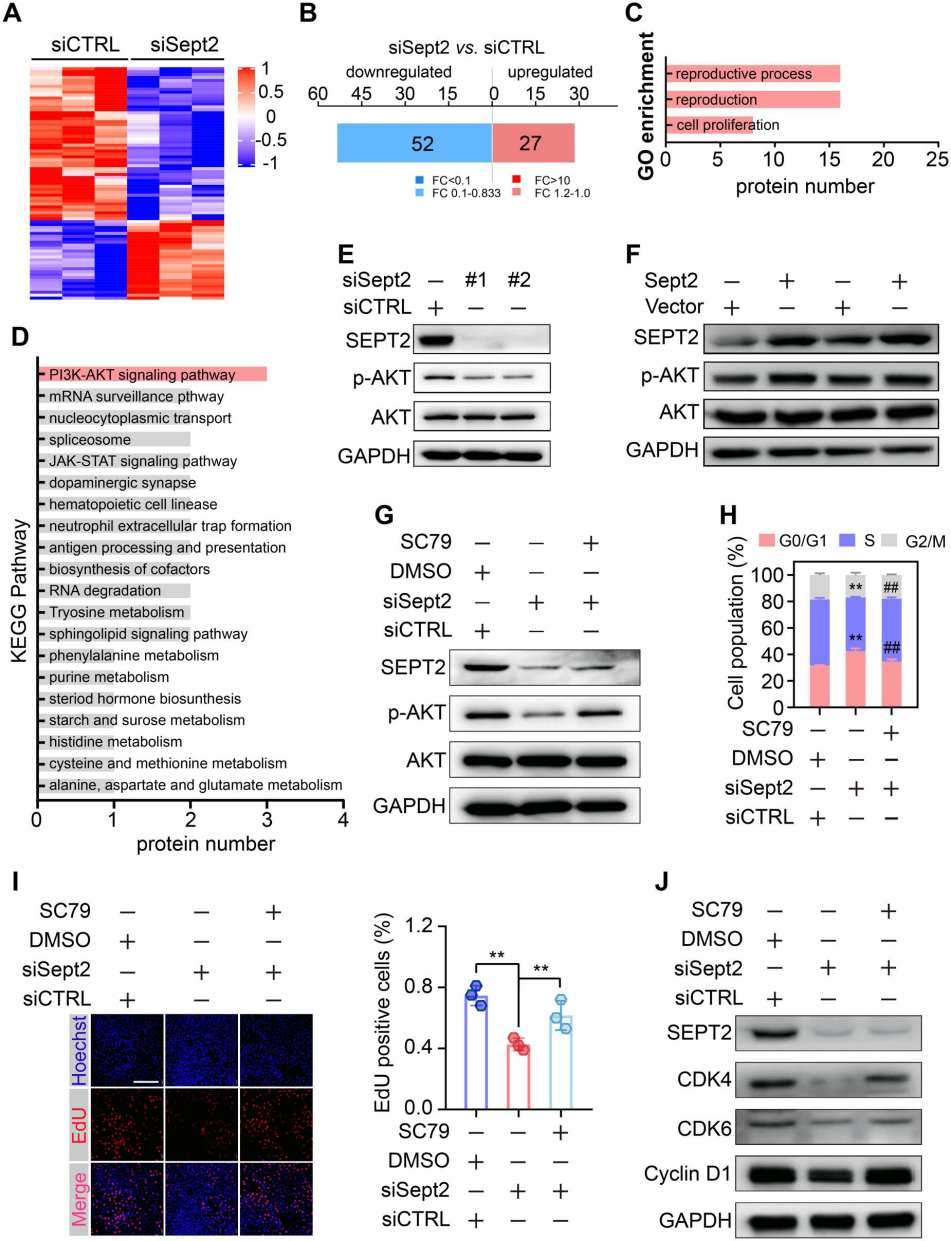
**Septin2 (*Sept2*) promoters spermatogonia proliferation *via* activation of the PI3K–AKT signaling pathway**. (**A**) Heatmap of the DEPs in GC-2 cells with *Sept2* knockdown. (**B**) DEPs in GC-2 cells with or without *Sept2* knockdown. (**C**) GO enrichment plot of the DEPs in GC-2 cells with or without *Sept2* knockdown. (**D**) KEGG enrichment plot of the DEPs in GC-2 cells with or without *Sept2* knockdown. (**E, F**) Protein levels of *Sept2*, AKT, and p-AKT in GC-2 cells with *Sept2* knockdown or overexpression. (**G**) Protein levels of *Sept2*, p-AKT, and AKT in *Sept2* abrogated GC-2 cells treated with or without SC79 (10 µM). (**H**) Cell cycle distribution in *Sept2* abrogated GC-2 cells treated with or without SC79 (10 µM). (**I**) Representative image of EdU incorporation assay in *Sept2* abrogated GC-2 cells treated with or without SC79 (10µM); scale bar = 100 µm. (**J**) Protein levels of SEPT2, CDK4, CDK6, and Cyclin D1 in *Sept2* abrogated GC-2 cells treated with or without SC79 (10 µM). Data were analyzed using unpaired Student’s *t*-test and presented as mean±SD. **P < 0.01. ##, The *P*-value of siSept2 with DMSO versus siSept2 with SC79 is less than 0.01. siCTRL, negative control siRNA; GO, Gene Ontology; KEGG, Kyoto Encyclopedia of Genes and Genomes; DEPs, differentially expressed proteins; FC, fold change; DMSO, dimethyl sulfoxide.

We performed TMT-based quantitative proteomic analysis on GC-2 cells with or without *Sept2* knockdown to elucidate how *Sept2* regulates spermatogonial proliferation (Fig. 6A). A total of 79 differentially expressed proteins (DEPs, fold change ≥1.2, *P* < 0.05), including 52 downregulated and 27 upregulated proteins, were identified and quantified (Fig. 6B). Notably, more than 20% of these DEPs (16/79) were involved in the ‘reproductive process’, and more than 10% (8/79) were involved in the ‘cell proliferation’ process, according to GO enrichment analysis (Fig. 6C), further demonstrating the essential role of *Sept2* in regulating spermatogonia proliferation and male reproduction. KEGG pathway analysis identified the PI3K–AKT signaling pathway as the most significantly enriched pathway following *Sept2* abrogation (Fig. 6D and Supplementary Fig. S9A). Western blotting confirmed that *Sept2* knockdown strikingly decreased AKT phosphorylation (Fig. 6E), whereas *Sept2* overexpression increased AKT phosphorylation levels in GC-2 cells (Fig. 6F). Notably, the reduced AKT phosphorylation level and impaired proliferation of GC-2 cells triggered by *Sept2* depletion were largely abolished in the presence of the AKT phosphorylation agonist SC79 (Fig. 6G–J), suggesting a crucial role for the AKT pathway in *Sept2*-mediated spermatogonia proliferation.

**Figure 7.**
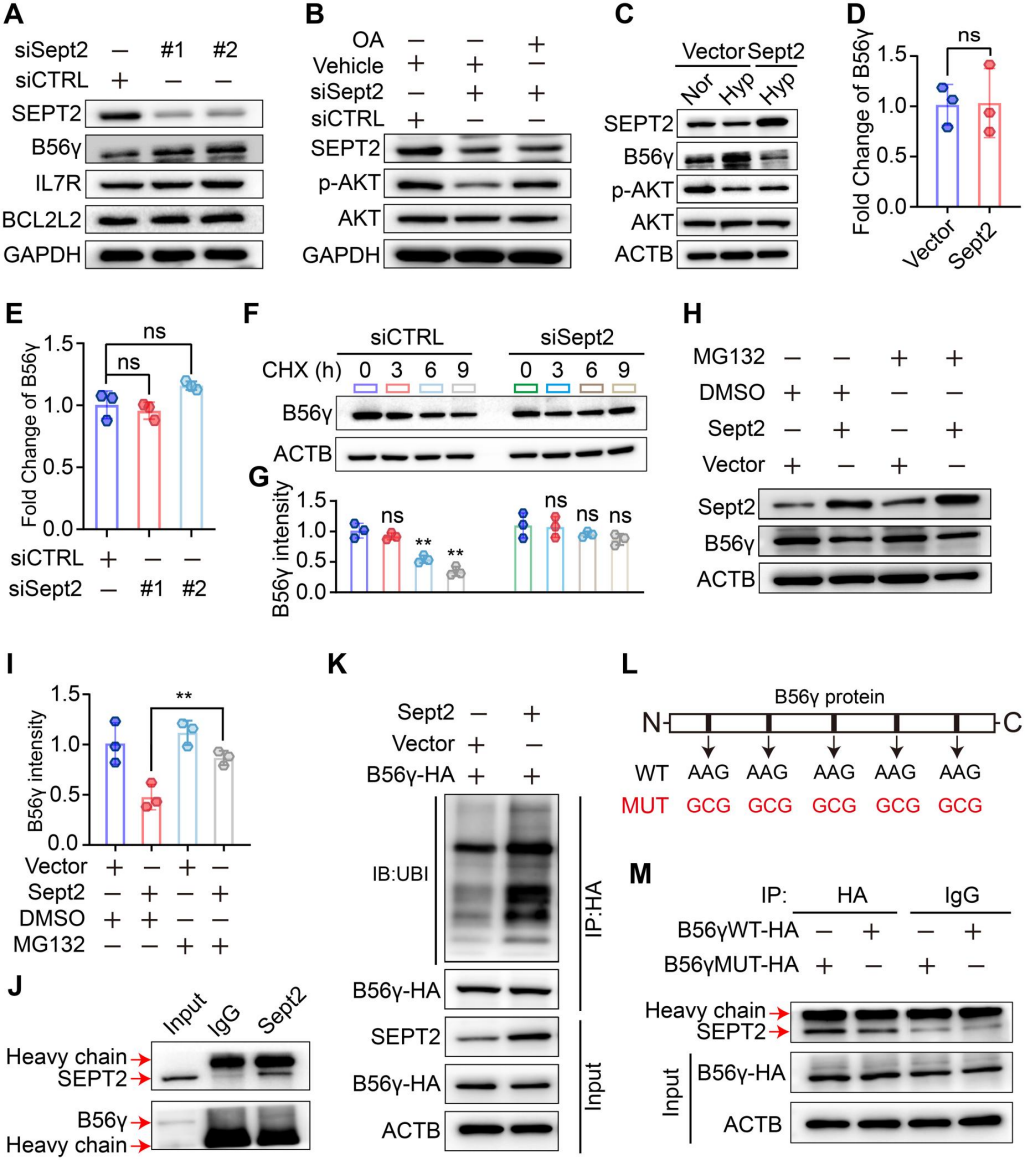
**Septin2 (*Sept2*) promotes B56γ ubiquitylation and degradation**. (**A**) Protein levels of SEPT2, B56γ, IL7R, and BCL2L2 in GC-2 cells with or without *Sept2* knockdown. (**B**) Protein levels of SEPT2, p-AKT, and AKT in *Sept2* abrogated GC-2 cells with or without OA (20 nM) treatment. (**C**) Protein levels of SEPT2, B56γ, p-AKT, and AKT in hypoxia-treated GC-2 cells with or without *Sept2* overexpression. (**D, E**) Relative mRNA level of B56γ in GC-2 cells with *Sept2* knockdown or overexpression. (**F, G**) Protein level and associated gray value analysis of B56γ in *Sept2* abrogated GC-2 cells after treatment with CHX (100 μg/ml) for different times. (**H, I**) Protein level and associated gray value analysis of B56γ in *Sept2* overexpressed GC-2 cells after treatment with MG132 (10 μM) for 6 h. (**J**) Co-immunoprecipitation (Co-IP)-immunoblotting showing the interaction between B56γ and SEPT2. (**K**) Co-IP-immunoblotting of the ubiquitinated B56γ in GC-2 cells with or without *Sept2* overexpression. (**L**) Schematic diagram of the mutation of the deubiquitinase binding sites in B56γ protein. (**M**) Co-IP-immunoblotting showing the interaction between B56γ and SEPT2 in wild-type or mutant B56γ-expressed GC-2 cells. Data were analyzed using unpaired Student’s *t*-test and presented as mean±SD. **P < 0.01. siCTRL, negative control siRNA; OA, okadaic acid; Nor, normoxia; Hyp, hypoxia; ns, not significant; WT, wild-type; MUT, mutant; CHX, cycloheximide; DMOG, Dimethyloxalylglycine.

Next, we determined how *Sept2* regulates AKT phosphorylation. By screening three proteins involved in the PI3K–AKT pathway, we found that the expression of PP2A regulatory subunit B56γ (also known as PPP2R5C) was markedly elevated following *Sept2* knockdown (Fig. 7A). PP2A is a direct phosphatase of AKT and can control G1–S progression through direct dephosphorylation of AKT *via* its subunit B56γ (Van Kanegan *et al.*, 2005; Rocher *et al.*, 2007; Sablina *et al.*, 2010). Treatment with the PP2A inhibitor OA significantly attenuated *Sept2* knockdown-mediated AKT dephosphorylation in GC-2 cells (Fig. 7B). Of note, we discovered that although hypoxia treatment increased B56γ expression and suppressed AKT phosphorylation in GC-2 cells, *Sept2* overexpression abolished these effects (Fig. 7C).

To better understand how *Sept2* regulates B56γ expression, we first quantified the mRNA levels of B56γ in GC-2 cells when *Sept2* was overexpressed or depleted. The results showed that *Sept2* abrogation or overexpression did not influence B56γ mRNA levels (Fig. 7D and E). Protein stability assays showed that *Sept2* abrogation alleviated the degradation of B56γ protein in the CHX-chased GC-2 cells (Fig. 7F and G). The ubiquitin-proteasome system represents one of the predominant post-transcriptional regulatory mechanisms governing protein expression during spermatogenesis (Xiong *et al.*, 2022), and latest evidence showed that *Sept2* participates in regulating protein ubiquitination and degradation (Fu *et al.*, 2023). We found that the ubiquitin-proteasome inhibitor MG132 mitigated the reduction of B56γ caused by *Sept2* overexpression (Fig. 7H and I). Using co-immunoprecipitation (Co-IP) followed by immunoblotting, we showed that *Sept2* physically interacts with B56γ and promotes its ubiquitination (Fig. 7J and K). This interaction was disrupted when the deubiquitinase binding sites in the B56γ protein were mutated (Fig. 7L and M), indicating that SEPT2 competes with the deubiquitinase for binding to B56γ. These results suggest that SEPT2 governs spermatogonial proliferation *via* PP2A-mediated AKT dephosphorylation.

### OA alleviated hypoxia-induced reproductive impairments in mice, while PP2A agonists aggravated these pathological effects

**Figure 8.**
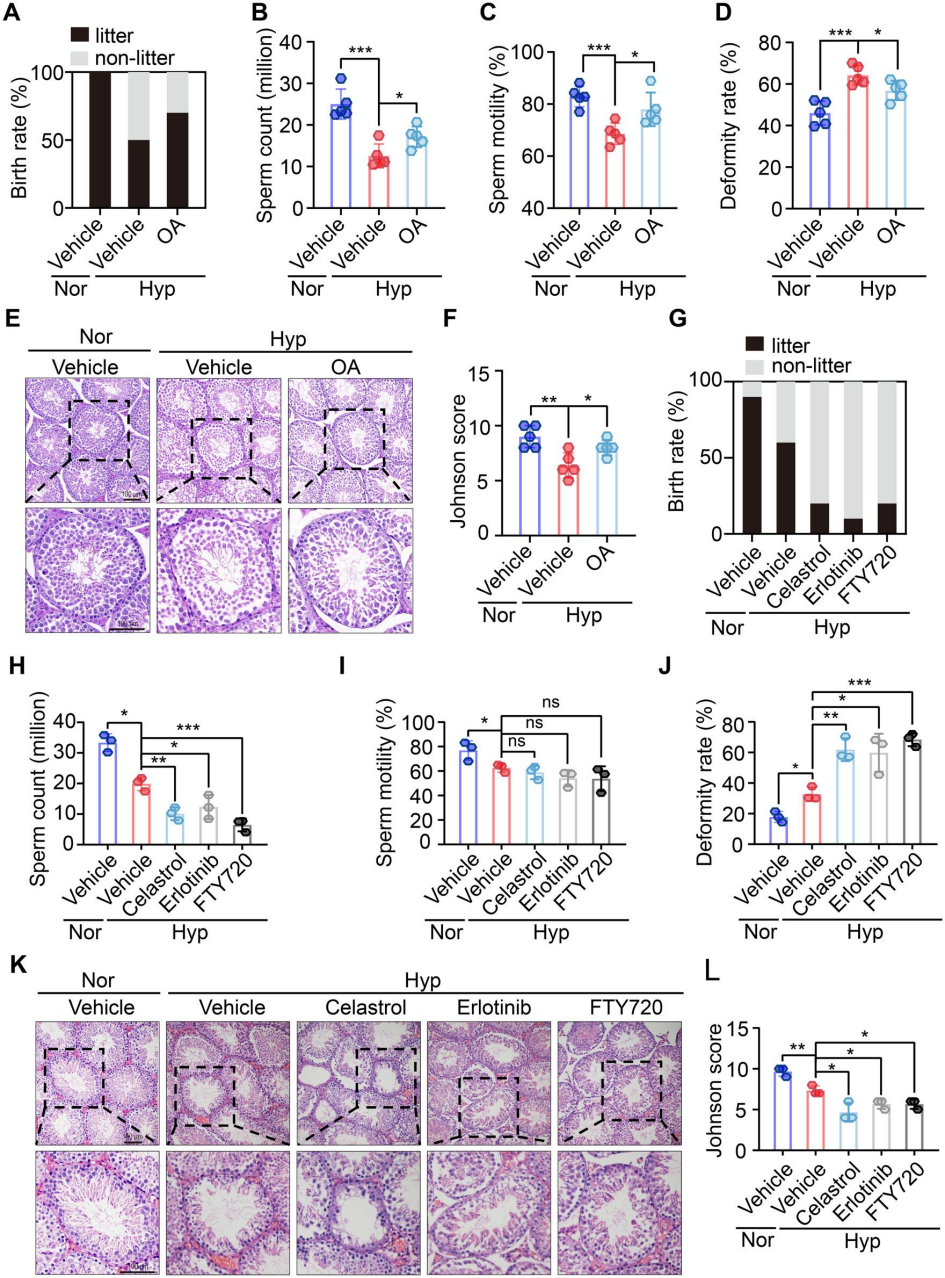
**Okadaic acid rescues hypoxia-induced male reproductive disorders, while protein phosphatase 2A (PP2A) agonists exacerbate these adverse effects in male mice**. (**A**) Male mice aged 6–8 weeks were treated with vehicle, vehicle with hypoxia, or OA with hypoxia. Fertility assessment showing the birth rate of female mice mated with males treated with vehicle, vehicle with hypoxia, or OA with hypoxia (n = 5 males and 10 females per group). (**B, C**) Sperm count and motility rate in mice treated with OA, as detected by CASA (n = 5 per group). (**D**) Sperm deformity rate in mice treated with vehicle, vehicle with hypoxia, or OA with hypoxia, as detected by eosin staining (n = 5 per group). (**E, F**) Representative images of H&E staining and relative Johnson score of testes from mice treated with vehicle, vehicle with hypoxia, and OA with hypoxia (n = 5 per group). Scale bars = 100 µm. (**G**) Fertility assessment showing the birth rate of female mice mated with Nor or Hyp males treated with different agonists (n = 3 males and 6 females per group). (**H, I**) Sperm count, and motility rate in Nor or Hyp males treated with different agonists, as detected by CASA (n = 3 per group). (**J**) Sperm deformity rate in Nor or Hyp males treated with different agonists, as detected by eosin staining (n = 3 per group). (**K, L**) Representative image of H&E staining and relative Johnson score of testes from Nor and Hyp males treated with different agonists (n = 3 per group). Scale bars = 100 µm. Data were analyzed using unpaired Student’s *t*-test and presented as mean±SD. *P < 0.05, **P < 0.01, ***P < 0.001. Nor, normoxia; Hyp, hypoxia; OA, okadaic acid; CASA, computer-assisted sperm analysis.

Until now, limited medications exist for treating male subfertility caused by hypoxia. Therefore, we explored the therapeutic potential of OA due to its proven ability to alleviate *Sept2* deficiency-mediated AKT dephosphorylation (Fig. 7B). Thus, we treated hypoxic mice with OA for 5 weeks and observed that OA treatment effectively improved the fertility rate, enhanced sperm quality, and reshaped the histoarchitecture of the seminiferous epithelium in hypoxic mice (Fig. 8A–F). These findings indicate the therapeutic potential of OA for hypoxia-induced male infertility.

Compelling evidence has revealed a strong correlation between PP2A dysfunction and a range of diseases commonly observed in individuals with hypoxia, including pulmonary arterial hypertension (Naeije *et al.*, 2022), multiple sclerosis (Ho *et al.*, 2012), inflammation (Eltzschig and Carmeliet, 2011), and cancer (Shi *et al.*, 2019). PP2A agonists have been widely used to treat these conditions. However, the reproductive effects of these treatments are largely unknown. We initially selected three clinically available PP2A agonists with a wide range of applications, celastrol, erlotinib, and FTY720 for reproductive investigation. Using a hypoxic mouse model, we revealed that treatment with celastrol, erlotinib, or FTY720 exacerbated the negative impact of hypoxia on male fertility, as evidenced by a reduced fertility rate (Fig. 8G), lower sperm quality (Fig. 8H–J), and worsened seminiferous epithelium (Fig. 8K and L).

Finally, we evaluated the reproductive toxicity of celastrol, erlotinib, and FTY720 in naïve male mice (under normoxic conditions). Celastrol, erlotinib, or FTY720 treatment reduced AKT phosphorylation level (Supplementary Fig. S9B), fertility rate (Supplementary Fig. S9C), sperm quality (Supplementary Fig. S9D–F), and histoarchitecture of the seminiferous epithelium (Supplementary Fig. S9G and H). These findings highlight the potential reproductive risks associated with PP2A agonists, particularly for men with reproductive intentions or those already affected by hypoxia.

## Discussion

**Figure 9.**
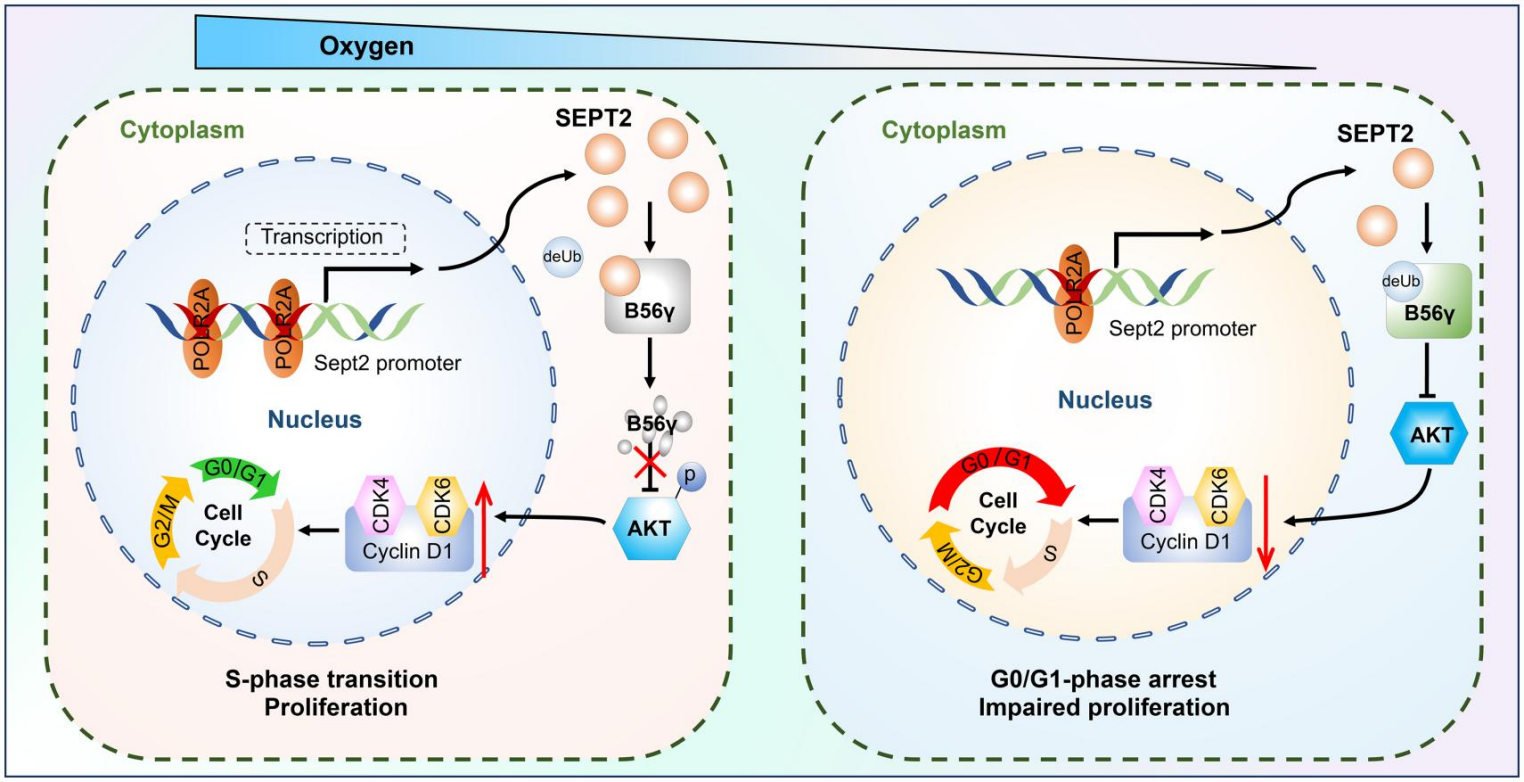
**Mechanisms by which hypoxia impairs spermatogonial proliferation. **Hypoxia exposure impairs spermatogonia proliferation *via* the SEPT2–PP2A/B56γ–AKT signaling pathway. Hypoxia inhibits the binding of POLR2A to the Septin2 (*Sept2*) promoter, thereby suppressing *Sept2* transcriptional activity. This downregulation of SEPT2 reduces its competition with the deubiquitinase responsible for PP2A/B56y degradation, leading to enhanced PP2A/B56y stability. As a result, PP2A-mediated AKT dephosphorylation triggers G1–S phase cell cycle arrest, ultimately impairing the proliferative capacity of spermatogonia.

This study highlights the deleterious influence of hypoxia on male fertility, with impaired sperm parameters in men exposed to HAs and PH. Using a hypoxia-induced mouse model, we have demonstrated that hypoxia led to deficient spermatogonial proliferation and *Sept2* deficiency. *Sept2* knockdown in normoxic mice resulted in decreased fertility, reduced sperm quality, and disrupted seminiferous epithelial architecture, mirroring the phenotypes observed in hypoxic mice. Mechanistically, SEPT2 promotes the ubiquitination and degradation of the PP2A regulatory subunit B56γ, thereby supporting spermatogonia proliferation by preventing PP2A/B56γ-mediated AKT dephosphorylation. Furthermore, hypoxia reduced *Sept2* transcription by impairing the interaction between POLR2A and the *Sept2* promoter (Fig. 9). Finally, we demonstrated that the PP2A inhibitor OA effectively alleviated hypoxia-induced reproductive impairments in mice, while PP2A agonists aggravated these pathological effects. These mechanistic insights not only advance the understanding of hypoxia-associated male reproductive dysfunction but also highlight OA as a promising therapeutic strategy for such conditions.

In recent decades, the influences of hypoxia stress on andrological health parameters have gained significant attention (Lord, 2025). Several longitudinal studies have consistently reported that men exposed to HA hypoxia showed significant declines in semen parameters, including decreased sperm concentration, impaired motility, and elevated morphological abnormalities (Verratti *et al.*, 2016, 2022; Xia *et al.*, 2024). Beyond sperm parameters, hypoxia also alters reproductive hormone profiles, with dysregulation in LH, testosterone, prolactin, and FSH levels (Okumura *et al.*, 2003; Pelliccione *et al.*, 2011; Verratti *et al.*, 2016). Disease-associated hypoxia occurring in conditions such as sickle cell disease (Osegbe *et al.*, 1981; Smith-Whitley, 2014), beta-thalassemia (Safarinejad, 2008), sleep apnea syndrome (Alvarenga *et al.*, 2023), chronic obstructive airway disease (Semple *et al.*, 1981), and idiopathic pulmonary fibrosis (Semple *et al.*, 1984), exhibits similar adverse effects on male reproductive health. In this study, we used a cross-sectional study to assess semen parameters in men exposed to HAs and those with PH, with parallel assessment conducted in a matched control cohort. Consistent with previous findings, individuals exposed to HA hypoxia exhibited significant reductions in sperm concentration, total sperm count, and motility, and increased sperm malformations. Beyond these parameters, we also evaluated semen volume, pH, liquefaction time, and sperm viability rate, and classified sperm malformations and motility types. Our findings showed no significant changes in semen volume or pH, but revealed reduced sperm viability, prolonged liquefaction time, and particular sensitivity of sperm PR motility and head morphology to hypoxic conditions. Interestingly, men with PH demonstrated markedly decreased PR and total motility. Although other semen parameters showed a worsening trend, the differences were not statistically significant. This finding could potentially be explained by the limited number of subjects in the PH cohort, which may have been insufficient to detect statistically significant differences in certain semen parameters. The observed trends, although not statistically significant, suggested a potential impact of hypoxia that could become more evident with a larger sample size.

Animal models that mimic hypoxia in young men provide a valuable approach for studying the functional and mechanistic aspects of male reproduction. Several hypoxia models have been developed, including hypoxic chamber-induced systemic hypoxia, experimental varicocele-induced testicular hypoxia, and HA hypoxia models (Torres *et al.*, 2014; Hassanin *et al.*, 2018; He *et al.*, 2021). Existing literature using these models has demonstrated that hypoxia negatively affects sperm quality, reproductive endocrinology, and spermatogenesis in rodents, primates, ruminants, and fish (Benso *et al.*, 2007; He *et al.*, 2015; Ayman *et al.*, 2021). Our investigation employed a hypoxic chamber model in which oxygen levels were reduced to half that of normoxia, to replicate the oxygen deprivation experienced by young males. Adult male mice were maintained in a hypoxic chamber for 35 days (a full spermatogenic cycle) to comprehensively analyze the impact of hypoxia on different stages of spermatogenesis. Consistent with previous reports, we observed impaired sperm count, motility, morphology, viability rate, and seminiferous epithelial architecture in hypoxic mice. Bulk RNA sequencing of testis samples revealed a deficiency in spermatogonial proliferation as a key pathological change associated with hypoxia-induced subfertility. However, this study did not assess reproductive hormones in either the clinical cohort or the hypoxic mice because the primary focus was on sperm parameters. Given the crucial role of reproductive endocrinology in regulating spermatogenesis and male fertility, as well as the well-documented hormone dysregulation under hypoxic conditions, future investigations are needed to explore hormonal alterations and their underlying mechanisms.

SEPT2 is a GTP-binding protein responsible for multiple biological functions, e.g. cytoskeletal organization, cell division, cell polarity, and signal transduction (Al-Ali *et al.*, 2024). Here, we revealed that SEPT2 is highly expressed in the testes, with a wide distribution across various spermatogenic cells. Previous studies have established SEPT2 as a fundamental component of the connecting structure in the sperm neck (Huang *et al.*, 2018; Shen *et al.*, 2020) and reported reduced *Sept2* transcript levels in sperm from asthenozoospermic patients (Mazaheri Moghaddam *et al.*, 2021). Spearman’s correlation analysis revealed a strong association between *SEPT2* expression and sperm motility in men, highlighting its potential role in regulating motility (Mazaheri Moghaddam *et al.*, 2021). Our findings revealed a significant reduction in *Sept2* expression in spermatogonia from hypoxic mice and confirmed its critical role in spermatogonial proliferation. The high evolutionary conservation of SEPT2, combined with its ability to rescue proliferation defects, suggests its potential as a viable treatment target for hypoxia-induced fertility impairment. Further mechanistic studies showed that SEPT2 interacts with and promotes the degradation of the B56γ component of the PP2A heterotrimeric complex. PP2A functions as a serine/threonine phosphatase, whose holoenzyme structure consists of three distinct components: a structural scaffold (A), an enzymatic core (C), and various regulatory (B) elements. The regulatory B subunit confers specificity and functional complexity to PP2A by determining its substrate recognition and subcellular localization (Peris *et al.*, 2023). Previous research has established PP2A as a direct phosphatase of AKT, regulating G1-S progression through B56γ-mediated AKT dephosphorylation (Rocher *et al.*, 2007). We found that hypoxia impairs the SEPT2–B56γ interaction by inhibiting *Sept2* transcription, which prevents B56γ degradation. This stabilization of B56γ enhances PP2A-mediated AKT dephosphorylation, leading to G1–S arrest in spermatogonia. Notably, the PP2A inhibitor OA showed therapeutic efficacy in alleviating hypoxia-induced subfertility in hypoxic mice.

This study highlights the reproductive toxicity of PP2A agonists. PP2A serves as a critical regulator in multiple physiological processes, encompassing immune responses, tumorigenesis, and neurobiology (Clark and Ohlmeyer, 2019; Fevga *et al.*, 2023; Peris *et al.*, 2023). Celastrol, erlotinib, and FTY720 are Food and Drug Administration (FDA)-approved PP2A agonists commonly used in clinical practice. Celastrol activates PP2A by promoting the degradation of CIP2A, an endogenous PP2A inhibitor, and is used in antioxidant, anti-inflammatory, and anticancer therapies (O’Connor *et al.*, 2018). Erlotinib transcriptionally downregulates CIP2A to upregulate PP2A and is primarily used to treat EGFR-mutant lung cancer (Liu *et al.*, 2017). FTY720 enhances PP2A activity by disrupting the interaction between PP2A and SET, an endogenous PP2A inhibitor, and is prescribed for managing multiple sclerosis (Pelletier and Hafler, 2012). However, their reproductive toxicity remains poorly understood. Our study found that exposure to celastrol, erlotinib, or FTY720 exacerbated hypoxia-induced deficiencies in spermatogonia proliferation, reduced sperm quality, and decreased fertility in male mice. Similarly, normoxic mice treated with these PP2A agonists displayed reproductive deterioration comparable to that in hypoxic or FCF-treated mice. These results suggest that men who intend to conceive, especially those experiencing hypoxia, should avoid PP2A agonists due to their potential reproductive toxicity.

HIFs consist of two subunits: an oxygen-sensitive α-subunit (HIF-1α) and a stably expressed β-subunit (HIF-1β), forming heterodimeric complexes. Under normoxic conditions, HIF-1α is hydroxylated on proline residues by prolyl hydroxylase domain enzymes (PHDs), leading to its ubiquitin–proteasome pathway-mediated destruction. In hypoxia, PHDs remain functionally inactive, permitting HIF-1α/HIF-1β dimer formation and subsequent binding to DNA sequences containing hypoxia-responsive elements motifs, regulating gene expression (Gonzalez *et al.*, 2018). While HIFs have traditionally been considered the primary mediators of hypoxia-regulated gene expression (Choudhry and Harris, 2018), recent studies have identified HIF-independent mechanisms. Dickson *et al.* demonstrated that the hypoxia-induced degradation of SREBP2 overrides the sterol-sensing response independent of HIFs (Dickson *et al.*, 2023). Similarly, Zhang *et al.* reported that hypoxia upregulates hemoglobin subunit genes (HBA and HBB) *via* the KLF1 rather than HIF pathways (Zhang *et al.*, 2023). Our research findings further substantiate the existing evidence base, showing that hypoxia inhibits *Sept2* transcription by disrupting the interaction between POLR2A and the *Sept2* promoter, independently of HIF-1α.

In addition to cell proliferation, RNA-seq analysis of testes identified apoptosis as one of the top enriched GO terms. Clinical and animal studies have consistently linked hypoxia to increased apoptosis in spermatogenic cells (Ghandehari-Alavijeh *et al.*, 2019; Cornejo-Guerra *et al.*, 2024). Hypoxia-induced apoptosis is multifactorial and involves HIF activation, which modulates pro-apoptotic proteins such as Bax and Caspase-3 while suppressing protective proteins such as BCL-2 (Ghandehari-Alavijeh *et al.*, 2019; Babaei *et al.*, 2022). Additionally, hypoxia-induced oxidative stress, characterized by elevated reactive oxygen species (ROS) levels, exacerbates mitochondrial dysfunction and activates apoptotic pathways (Zepeda *et al.*, 2014; Li *et al.*, 2025). Using TUNEL assays, IHC staining, and western blotting, we observed increased DNA fragmentation and cleaved-Caspase 3 expression in spermatogenic cells from hypoxic mice (Supplementary Fig. S3A–C). Elevated levels of MDA, a marker of oxidative stress, were also detected in hypoxic testes (Supplementary Fig. S3D). These findings, along with previous reports, demonstrate that excessive apoptosis and oxidative stress contribute to the hypoxia-induced reproductive decline.

Overall, this study provides complementary human and animal study data establishing hypoxic impairment of spermatogenic function and offers mechanistic insights into the underlying processes. We demonstrated that hypoxia impairs *Sept2* transcription, disrupting the SEPT2–B56γ interaction and preventing B56γ degradation. This enhances PP2A/B56γ-mediated AKT dephosphorylation, leading to G1–S arrest in spermatogonia and reduced its proliferation. The PP2A inhibitor OA was found to effectively mitigate hypoxia-induced reproductive decline and improve sperm parameters, fertility rates, and seminiferous epithelium architecture in hypoxic mice. Conversely, PP2A agonists exacerbate hypoxia-induced reproductive damage.

Our findings highlight that the SEPT2–PP2A/B56γ–AKT axis could serve as a promising target for treating hypoxia-related infertility, with potential applications in reproductive medicine for individuals affected by hypoxia.

## Supplementary Material

hoaf027_Supplementary_Data

## Data Availability

The datasets generated and/or analyzed during the current study are available at the China National Center for Bioinformation (https://www.cncb.ac.cn/), under Project PRJCA035733.

## References

[hoaf027-B1] Adler ID. Comparison of the duration of spermatogenesis between male rodents and humans. Mutat Res 1996;352:169–172. 8676906 10.1016/0027-5107(95)00223-5

[hoaf027-B2] Agarwal A , Baskaran S, Parekh N, Cho CL, Henkel R, Vij S, Arafa M, Panner Selvam MK, Shah R. Male infertility. Lancet 2021;397:319–333. 33308486 10.1016/S0140-6736(20)32667-2

[hoaf027-B3] Al-Ali H , Baig A, Alkhanjari RR, Murtaza ZF, Alhajeri MM, Elbahrawi R, Abdukadir A, Bhamidimarri PM, Kashir J, Hamdan H. Septins as key players in spermatogenesis, fertilisation and pre-implantation embryogenic cytoplasmic dynamics. Cell Commun Signal 2024;22:523. 39468561 10.1186/s12964-024-01889-z PMC11514797

[hoaf027-B4] Alvarenga TA , Fernandes GL, Bittencourt LR, Tufik S, Andersen ML. The effects of sleep deprivation and obstructive sleep apnea syndrome on male reproductive function: a multi-arm randomised trial. J Sleep Res 2023;32:e13664. 35670262 10.1111/jsr.13664

[hoaf027-B5] Ashburner M , Ball CA, Blake JA, Botstein D, Butler H, Cherry JM, Davis AP, Dolinski K, Dwight SS, Eppig JT et al Gene ontology: tool for the unification of biology. The Gene Ontology Consortium. Nat Genet 2000;25:25–29. 10802651 10.1038/75556 PMC3037419

[hoaf027-B6] Ayman AH , Anas AO, Yasser AA, Masad AM, Abdullah AS, Mazen A, Salih AG, Saad AS, Abdulraheem A, AbdulRhman AG et al Levels of follicle stimulating hormone (FSH), luteinizing hormone (LH), testosterone and prolactin at moderate altitude inhabitant male. Pak J Biol Sci 2021;24:188–192. 33683047 10.3923/pjbs.2021.188.192

[hoaf027-B7] Babaei A , Moradi S, Hoseinkhani Z, Rezazadeh D, Dokaneheifard S, Asadpour R, Sharma G, Mansouri K. Expression of hypoxia-inducible factor1-alpha in varicocele disease: a comprehensive systematic review. Reprod Sci 2022;29:2731–2743. 34313997 10.1007/s43032-021-00696-y

[hoaf027-B8] Benso A , Broglio F, Aimaretti G, Lucatello B, Lanfranco F, Ghigo E, Grottoli S. Endocrine and metabolic responses to extreme altitude and physical exercise in climbers. Eur J Endocrinol 2007;157:733–740. 18057380 10.1530/EJE-07-0355

[hoaf027-B9] Chen S. Ultrafast one-pass FASTQ data preprocessing, quality control, and deduplication using fastp. Imeta 2023;2:e107. 38868435 10.1002/imt2.107 PMC10989850

[hoaf027-B10] Choudhry H , Harris AL. Advances in hypoxia-inducible factor biology. Cell Metab 2018;27:281–298. 29129785 10.1016/j.cmet.2017.10.005

[hoaf027-B11] Clark AR , Ohlmeyer M. Protein phosphatase 2A as a therapeutic target in inflammation and neurodegeneration. Pharmacol Ther 2019;201:181–201. 31158394 10.1016/j.pharmthera.2019.05.016 PMC6700395

[hoaf027-B12] Cornejo-Guerra C , Salazar-Ardiles C, Morales P, Andrade DC. Consequences of exposure to hypobaric hypoxia associated with high altitude on spermatogenesis and seminal parameters: a literature review. Cells 2024;13:592. 38607031 10.3390/cells13070592 PMC11011536

[hoaf027-B13] Di Persio S , Tekath T, Siebert-Kuss LM, Cremers JF, Wistuba J, Li X, Meyer Zu Horste G, Drexler HCA, Wyrwoll MJ, Tuttelmann F et al Single-cell RNA-seq unravels alterations of the human spermatogonial stem cell compartment in patients with impaired spermatogenesis. Cell Rep Med 2021;2:100395. 34622232 10.1016/j.xcrm.2021.100395 PMC8484693

[hoaf027-B14] Dickson AS , Pauzaite T, Arnaiz E, Ortmann BM, West JA, Volkmar N, Martinelli AW, Li Z, Wit N, Vitkup D et al A HIF independent oxygen-sensitive pathway for controlling cholesterol synthesis. Nat Commun 2023;14:4816. 37558666 10.1038/s41467-023-40541-1 PMC10412576

[hoaf027-B15] Eisenberg ML , Esteves SC, Lamb DJ, Hotaling JM, Giwercman A, Hwang K, Cheng YS. Male infertility. Nat Rev Dis Primers 2023;9:49. 37709866 10.1038/s41572-023-00459-w

[hoaf027-B16] Eltzschig HK , Carmeliet P. Hypoxia and inflammation. N Engl J Med 2011;364:656–665. 21323543 10.1056/NEJMra0910283 PMC3930928

[hoaf027-B17] Fevga C , Tesson C, Carreras Mascaro A, Courtin T, van Coller R, Sakka S, Ferraro F, Farhat N, Bardien S, Damak M et al; International Parkinsonism Genetics Network. PTPA variants and impaired PP2A activity in early-onset parkinsonism with intellectual disability. Brain 2023;146:1496–1510. 36073231 10.1093/brain/awac326 PMC10115167

[hoaf027-B18] FlowJo™ for Windows [computer software]. Version 10.8. Ashland, OR: Becton, Dickinson and Company, 2023.

[hoaf027-B19] Fu B , Xiong Y, Sha Z, Xue W, Xu B, Tan S, Guo D, Lin F, Wang L, Ji J et al SEPTIN2 suppresses an IFN-gamma-independent, proinflammatory macrophage activation pathway. Nat Commun 2023;14:7441. 37978190 10.1038/s41467-023-43283-2 PMC10656488

[hoaf027-B20] Gat Y , Zukerman Z, Chakraborty J, Gornish M. Varicocele, hypoxia and male infertility. Fluid mechanics analysis of the impaired testicular venous drainage system. Hum Reprod 2005;20:2614–2619. 15932914 10.1093/humrep/dei089

[hoaf027-B21] Gatterer H , Villafuerte FC, Ulrich S, Bhandari SS, Keyes LE, Burtscher M. Altitude illnesses. Nat Rev Dis Primers 2024;10:43. 38902312 10.1038/s41572-024-00526-w

[hoaf027-B22] Ghandehari-Alavijeh R , Tavalaee M, Zohrabi D, Foroozan-Broojeni S, Abbasi H, Nasr-Esfahani MH. Hypoxia pathway has more impact than inflammation pathway on etiology of infertile men with varicocele. Andrologia 2019;51:e13189. 30474123 10.1111/and.13189

[hoaf027-B23] Gonzalez FJ , Xie C, Jiang C. The role of hypoxia-inducible factors in metabolic diseases. Nat Rev Endocrinol 2018;15:21–32. 30275460 10.1038/s41574-018-0096-z PMC6624429

[hoaf027-B25] Guo J , Grow EJ, Mlcochova H, Maher GJ, Lindskog C, Nie X, Guo Y, Takei Y, Yun J, Cai L et al The adult human testis transcriptional cell atlas. Cell Res 2018;28:1141–1157. 30315278 10.1038/s41422-018-0099-2 PMC6274646

[hoaf027-B26] Guo J , Nie X, Giebler M, Mlcochova H, Wang Y, Grow EJ, Kim R, Tharmalingam M, Matilionyte G, Lindskog C et al; DonorConnect. The dynamic transcriptional cell atlas of testis development during human puberty. Cell Stem Cell 2020;26:262–276.e4. 31928944 10.1016/j.stem.2019.12.005 PMC7298616

[hoaf027-B27] Hassanin AM , Ahmed HH, Kaddah AN. A global view of the pathophysiology of varicocele. Andrology 2018;6:654–661. 29978951 10.1111/andr.12511

[hoaf027-B28] He J , Cui J, Wang R, Gao L, Gao X, Yang L, Zhang Q, Cao J, Yu W. Exposure to hypoxia at high altitude (5380 m) for 1 year induces reversible effects on semen quality and serum reproductive hormone levels in young male adults. High Alt Med Biol 2015;16:216–222. 26288097 10.1089/ham.2014.1046

[hoaf027-B29] He T , Guo H, Shen X, Wu X, Xia L, Jiang X, Xu Y, Chen D, Zhang Y, Tan D et al Hypoxia-induced alteration of RNA modifications in the mouse testis and sperm. Biol Reprod 2021;105:1171–1178. 34296257 10.1093/biolre/ioab142

[hoaf027-B30] Ho SL , Alappat L, Awad AB. Vitamin D and multiple sclerosis. Crit Rev Food Sci Nutr 2012;52:980–987. 22823346 10.1080/10408398.2010.516034

[hoaf027-B31] Hornbeck PV , Zhang B, Murray B, Kornhauser JM, Latham V, Skrzypek E. PhosphoSitePlus, 2014: mutations, PTMs and recalibrations. Nucleic Acids Res 2015;43:D512–D520. 25514926 10.1093/nar/gku1267 PMC4383998

[hoaf027-B32] Hu H , Miao YR, Jia LH, Yu QY, Zhang Q, Guo AY. AnimalTFDB 3.0: a comprehensive resource for annotation and prediction of animal transcription factors. Nucleic Acids Res 2019;47:D33–D38. 30204897 10.1093/nar/gky822 PMC6323978

[hoaf027-B33] Huang CY , Wang YY, Chen YL, Chen MF, Chiang HS, Kuo PL, Lin YH. CDC42 negatively regulates testis-specific SEPT12 polymerization. Int J Mol Sci 2018;19:2627. 30189608 10.3390/ijms19092627 PMC6163814

[hoaf027-B34] James ER , Carrell DT, Aston KI, Jenkins TG, Yeste M, Salas-Huetos A. The role of the epididymis and the contribution of epididymosomes to mammalian reproduction. Int J Mol Sci 2020;21:5377. 32751076 10.3390/ijms21155377 PMC7432785

[hoaf027-B35] Kanehisa M , Goto S, Sato Y, Furumichi M, Tanabe M. KEGG for integration and interpretation of large-scale molecular data sets. Nucleic Acids Res 2012;40:D109–D114. 22080510 10.1093/nar/gkr988 PMC3245020

[hoaf027-B36] Kremer BE , Adang LA, Macara IG. Septins regulate actin organization and cell-cycle arrest through nuclear accumulation of NCK mediated by SOCS7. Cell 2007;130:837–850. 17803907 10.1016/j.cell.2007.06.053 PMC2085444

[hoaf027-B37] Li W , Yin X, Zhang L. FOXA2 regulates endoplasmic reticulum stress, oxidative stress, and apoptosis in spermatogonial cells by the Nrf2 pathway under hypoxic conditions. Exp Cell Res 2025;444:114388. 39701358 10.1016/j.yexcr.2024.114388

[hoaf027-B38] Li Z , Wang S, Gong C, Hu Y, Liu J, Wang W, Chen Y, Liao Q, He B, Huang Y et al Effects of environmental and pathological hypoxia on male fertility. Front Cell Dev Biol 2021;9:725933. 34589489 10.3389/fcell.2021.725933 PMC8473802

[hoaf027-B39] Liu CY , Huang TT, Huang CT, Hu MH, Wang DS, Wang WL, Tsai WC, Lee CH, Lau KY, Yang HP et al EGFR-independent Elk1/CIP2A signalling mediates apoptotic effect of an erlotinib derivative TD52 in triple-negative breast cancer cells. Eur J Cancer 2017;72:112–123. 28027514 10.1016/j.ejca.2016.11.012

[hoaf027-B40] Lord T. Pathophysiological effects of hypoxia on testis function and spermatogenesis. Nat Rev Urol 2025; Epub ahead of print. https://doi.org/10.1038/s41585-024-00969-6 10.1038/s41585-024-00969-6 39762391

[hoaf027-B41] Lord T , Nixon B. Metabolic changes accompanying spermatogonial stem cell differentiation. Dev Cell 2020;52:399–411. 32097651 10.1016/j.devcel.2020.01.014

[hoaf027-B43] Mazaheri Moghaddam M , Mazaheri Moghaddam M, Amini M, Bahramzadeh B, Baghbanzadeh A, Biglari A, Sakhinia E. Evaluation of SEPT2 and SEPT4 transcript contents in spermatozoa from men with asthenozoospermia and teratozoospermia. Health Sci Rep 2021;4:e436. 34849407 10.1002/hsr2.436 PMC8611181

[hoaf027-B44] Michalski A , Damoc E, Hauschild JP, Lange O, Wieghaus A, Makarov A, Nagaraj N, Cox J, Mann M, Horning S. Mass spectrometry-based proteomics using Q Exactive, a high-performance benchtop quadrupole Orbitrap mass spectrometer. Mol Cell Proteomics 2011;10:M111.011015. 10.1074/mcp.M111.011015 PMC3284220 21642640

[hoaf027-B45] Minas A , Mahmoudabadi S, Gamchi NS, Antoniassi MP, Alizadeh A, Bertolla RP. Testicular torsion in vivo models: mechanisms and treatments. Andrology 2023;11:1267–1285. 36825607 10.1111/andr.13418

[hoaf027-B46] Minhas S , Bettocchi C, Boeri L, Capogrosso P, Carvalho J, Cilesiz NC, Cocci A, Corona G, Dimitropoulos K, Gul M et al; EAU Working Group on Male Sexual and Reproductive Health. European Association of Urology Guidelines on Male Sexual and Reproductive Health: 2021 update on male infertility. Eur Urol 2021;80:603–620. 34511305 10.1016/j.eururo.2021.08.014

[hoaf027-B47] Munzel T , Sorensen M, Hahad O, Nieuwenhuijsen M, Daiber A. The contribution of the exposome to the burden of cardiovascular disease. Nat Rev Cardiol 2023;20:651–669. 37165157 10.1038/s41569-023-00873-3

[hoaf027-B48] Naeije R , Richter MJ, Rubin LJ. The physiological basis of pulmonary arterial hypertension. Eur Respir J 2022;59:2102334. 34737219 10.1183/13993003.02334-2021 PMC9203839

[hoaf027-B49] Nathan SD , Barbera JA, Gaine SP, Harari S, Martinez FJ, Olschewski H, Olsson KM, Peacock AJ, Pepke-Zaba J, Provencher S et al Pulmonary hypertension in chronic lung disease and hypoxia. Eur Respir J 2019;53:1801914. 30545980 10.1183/13993003.01914-2018 PMC6351338

[hoaf027-B50] O’Connor CM , Perl A, Leonard D, Sangodkar J, Narla G. Therapeutic targeting of PP2A. Int J Biochem Cell Biol 2018;96:182–193. 29107183 10.1016/j.biocel.2017.10.008 PMC5927617

[hoaf027-B51] Okumura A , Fuse H, Kawauchi Y, Mizuno I, Akashi T. Changes in male reproductive function after high altitude mountaineering. High Alt Med Biol 2003;4:349–353. 14561240 10.1089/152702903769192304

[hoaf027-B52] Ortega FB , Lavie CJ, Blair SN. Obesity and cardiovascular disease. Circ Res 2016;118:1752–1770. 27230640 10.1161/CIRCRESAHA.115.306883

[hoaf027-B53] Osegbe DN , Akinyanju O, Amaku EO. Fertility in males with sickle cell disease. Lancet 1981;2:275–276. 6114323 10.1016/s0140-6736(81)90525-0

[hoaf027-B54] Pelletier D , Hafler DA. Fingolimod for multiple sclerosis. N Engl J Med 2012;366:339–347. 22276823 10.1056/NEJMct1101691

[hoaf027-B55] Pelliccione F , Verratti V, D’Angeli A, Micillo A, Doria C, Pezzella A, Iacutone G, Francavilla F, Di Giulio C, Francavilla S. Physical exercise at high altitude is associated with a testicular dysfunction leading to reduced sperm concentration but healthy sperm quality. Fertil Steril 2011;96:28–33. 21561607 10.1016/j.fertnstert.2011.03.111

[hoaf027-B56] Peris I , Romero-Murillo S, Vicente C, Narla G, Odero MD. Regulation and role of the PP2A-B56 holoenzyme family in cancer. Biochim Biophys Acta Rev Cancer 2023;1878:188953. 37437699 10.1016/j.bbcan.2023.188953

[hoaf027-B57] Perkins DN , Pappin DJ, Creasy DM, Cottrell JS. Probability-based protein identification by searching sequence databases using mass spectrometry data. Electrophoresis 1999;20:3551–3567. 10612281 10.1002/(SICI)1522-2683(19991201)20:18<3551::AID-ELPS3551>3.0.CO;2-2

[hoaf027-B58] Proteome Discoverer™ [computer program]. Version 1.4. Waltham, MA: Thermo Fisher Scientific, 2023.

[hoaf027-B59] Rabbani M , Zheng X, Manske GL, Vargo A, Shami AN, Li JZ, Hammoud SS. Decoding the spermatogenesis program: new insights from transcriptomic analyses. Annu Rev Genet 2022;56:339–368. 36070560 10.1146/annurev-genet-080320-040045 PMC10722372

[hoaf027-B60] Regmi S , Raut PK, Pathak S, Shrestha P, Park PH, Jeong JH. Enhanced viability and function of mesenchymal stromal cell spheroids is mediated via autophagy induction. Autophagy 2021;17:2991–3010. 33206581 10.1080/15548627.2020.1850608 PMC8526044

[hoaf027-B61] Richalet JP , Hermand E, Lhuissier FJ. Cardiovascular physiology and pathophysiology at high altitude. Nat Rev Cardiol 2024;21:75–88. 37783743 10.1038/s41569-023-00924-9

[hoaf027-B62] Rocher G , Letourneux C, Lenormand P, Porteu F. Inhibition of B56-containing protein phosphatase 2As by the early response gene IEX-1 leads to control of Akt activity. J Biol Chem 2007;282:5468–5477. 17200115 10.1074/jbc.M609712200

[hoaf027-B63] Sablina AA , Hector M, Colpaert N, Hahn WC. Identification of PP2A complexes and pathways involved in cell transformation. Cancer Res 2010;70:10474–10484. 21159657 10.1158/0008-5472.CAN-10-2855 PMC3056544

[hoaf027-B64] Safarinejad MR. Evaluation of semen quality, endocrine profile and hypothalamus-pituitary-testis axis in male patients with homozygous beta-thalassemia major. J Urol 2008;179:2327–2332. 18423706 10.1016/j.juro.2008.01.103

[hoaf027-B65] Semple PD , Beastall GH, Brown TM, Stirling KW, Mills RJ, Watson WS. Sex hormone suppression and sexual impotence in hypoxic pulmonary fibrosis. Thorax 1984;39:46–51. 6695352 10.1136/thx.39.1.46 PMC459720

[hoaf027-B66] Semple PD , Beastall GH, Watson WS, Hume R. Hypothalamic-pituitary dysfunction in respiratory hypoxia. Thorax 1981;36:605–609. 6797086 10.1136/thx.36.8.605 PMC471646

[hoaf027-B67] Shah S , Valiani D, Balogun O, Zanoria MA, Jarrett S, Hiedra R, Patarroyo-Aponte G, Azmaiparashvili Z, Lo KB, Eiger G. Demographic and clinical profile of patients suffering prolonged severe hypoxia in COVID-19. Expert Rev Respir Med 2022;16:1017–1021. 36122195 10.1080/17476348.2022.2126354

[hoaf027-B68] Shen YR , Wang HY, Tsai YC, Kuo YC, Wu SR, Wang CY, Kuo PL. The SEPT12 complex is required for the establishment of a functional sperm head-tail junction. Mol Hum Reprod 2020;26:402–412. 32392324 10.1093/molehr/gaaa031

[hoaf027-B69] Shi J , Yu T, Song K, Du S, He S, Hu X, Li X, Li H, Dong S, Zhang Y et al Dexmedetomidine ameliorates endotoxin-induced acute lung injury in vivo and in vitro by preserving mitochondrial dynamic equilibrium through the HIF-1a/HO-1 signaling pathway. Redox Biol 2021;41:101954. 33774474 10.1016/j.redox.2021.101954 PMC8027777

[hoaf027-B70] Shi Y , Fan S, Wu M, Zuo Z, Li X, Jiang L, Shen Q, Xu P, Zeng L, Zhou Y et al YTHDF1 links hypoxia adaptation and non-small cell lung cancer progression. Nat Commun 2019;10:4892. 31653849 10.1038/s41467-019-12801-6 PMC6814821

[hoaf027-B71] Smith-Whitley K. Reproductive issues in sickle cell disease. Blood 2014;124:3538–3543. 25472967 10.1182/blood-2014-07-577619

[hoaf027-B72] Torres M , Laguna-Barraza R, Dalmases M, Calle A, Pericuesta E, Montserrat JM, Navajas D, Gutierrez-Adan A, Farre R. Male fertility is reduced by chronic intermittent hypoxia mimicking sleep apnea in mice. Sleep 2014;37:1757–1765. 25364071 10.5665/sleep.4166 PMC4196059

[hoaf027-B73] Trapnell C , Roberts A, Goff L, Pertea G, Kim D, Kelley DR, Pimentel H, Salzberg SL, Rinn JL, Pachter L. Differential gene and transcript expression analysis of RNA-seq experiments with TopHat and Cufflinks. Nat Protoc 2012;7:562–578. 22383036 10.1038/nprot.2012.016 PMC3334321

[hoaf027-B74] UniProt Consortium. UniProt: the universal protein knowledgebase in 2021. Nucleic Acids Res 2021;49:D480–D489. 33237286 10.1093/nar/gkaa1100 PMC7778908

[hoaf027-B75] Van Kanegan MJ , Adams DG, Wadzinski BE, Strack S. Distinct protein phosphatase 2A heterotrimers modulate growth factor signaling to extracellular signal-regulated kinases and Akt. J Biol Chem 2005;280:36029–36036. 16129692 10.1074/jbc.M506986200

[hoaf027-B76] Verratti V , Di Giulio C, D’Angeli A, Tafuri A, Francavilla S, Pelliccione F. Sperm forward motility is negatively affected by short-term exposure to altitude hypoxia. Andrologia 2016;48:800–806. 26762696 10.1111/and.12515

[hoaf027-B77] Verratti V , Mrakic-Sposta S, Fusi J, Sabovic I, Franzoni F, Pietrangelo T, Bondi D, Dall’Acqua S, Daniele S, Scarfo G et al Fertility impairment after trekking at high altitude: a proof of mechanisms on redox and metabolic seminal changes. Int J Mol Sci 2022;23:9066. 36012330 10.3390/ijms23169066 PMC9409093

[hoaf027-B78] Wang C , Mbizvo M, Festin MP, Björndahl L, Toskin I; Other Editorial Board Members of the WHO Laboratory Manual for the Examination and Processing of Human Semen. Evolution of the WHO “Semen” processing manual from the first (1980) to the sixth edition (2021). Fertil Steril 2022;117:237–245. 34996596 10.1016/j.fertnstert.2021.11.037 PMC8842884

[hoaf027-B79] Wang C , Tan X, Tang D, Gou Y, Han C, Ning W, Lin S, Zhang W, Chen M, Peng D et al GPS-Uber: a hybrid-learning framework for prediction of general and E3-specific lysine ubiquitination sites. Brief Bioinform 2022;23:bbab574. 35037020 10.1093/bib/bbab574

[hoaf027-B80] Wang M , Liu X, Chang G, Chen Y, An G, Yan L, Gao S, Xu Y, Cui Y, Dong J et al Single-cell RNA sequencing analysis reveals sequential cell fate transition during human spermatogenesis. Cell Stem Cell 2018;23:599–614.e4. 30174296 10.1016/j.stem.2018.08.007

[hoaf027-B81] World Health Organization. WHO Laboratory Manual for the Examination and Processing of Human Semen, 5th ed. Geneva: World Health Organization, 2010.

[hoaf027-B82] Xia K , Luo P, Yu J, He S, Dong L, Gao F, Chen X, Ye Y, Gao Y, Ma Y et al Single-cell RNA sequencing reveals transcriptomic landscape and potential targets for human testicular ageing. Hum Reprod 2024;39:2189–2209. 39241251 10.1093/humrep/deae199 PMC11447013

[hoaf027-B83] Xiong Y , Yu C, Zhang Q. Ubiquitin-proteasome system-regulated protein degradation in spermatogenesis. Cells 2022;11:1058. 35326509 10.3390/cells11061058 PMC8947704

[hoaf027-B84] Yu B , Wang X, Song Y, Xie G, Jiao S, Shi L, Cao X, Han X, Qu A. The role of hypoxia-inducible factors in cardiovascular diseases. Pharmacol Ther 2022;238:108186. 35413308 10.1016/j.pharmthera.2022.108186

[hoaf027-B85] Zepeda AB , Figueroa CA, Calaf GM, Farias JG. Male reproductive system and antioxidants in oxidative stress induced by hypobaric hypoxia. Andrologia 2014;46:1–8. 23127143 10.1111/and.12039

[hoaf027-B86] Zhang F , Zhang B, Wang Y, Jiang R, Liu J, Wei Y, Gao X, Zhu Y, Wang X, Sun M et al An extra-erythrocyte role of haemoglobin body in chondrocyte hypoxia adaption. Nature 2023;622:834–841. 37794190 10.1038/s41586-023-06611-6 PMC10600011

