# Supplementary material for: Hypoxia exposure impairs male fertility via inhibiting Septin2-mediated spermatogonial proliferation
Source: Hum Reprod Open. 2025 May 14;2025(3):hoaf027. doi: 10.1093/hropen/hoaf027 (PMC12145212; doi:10.1093/hropen/hoaf027)
Supplement: hoaf027_Supplementary_Data [file hoaf027_supplementary_data.pdf]

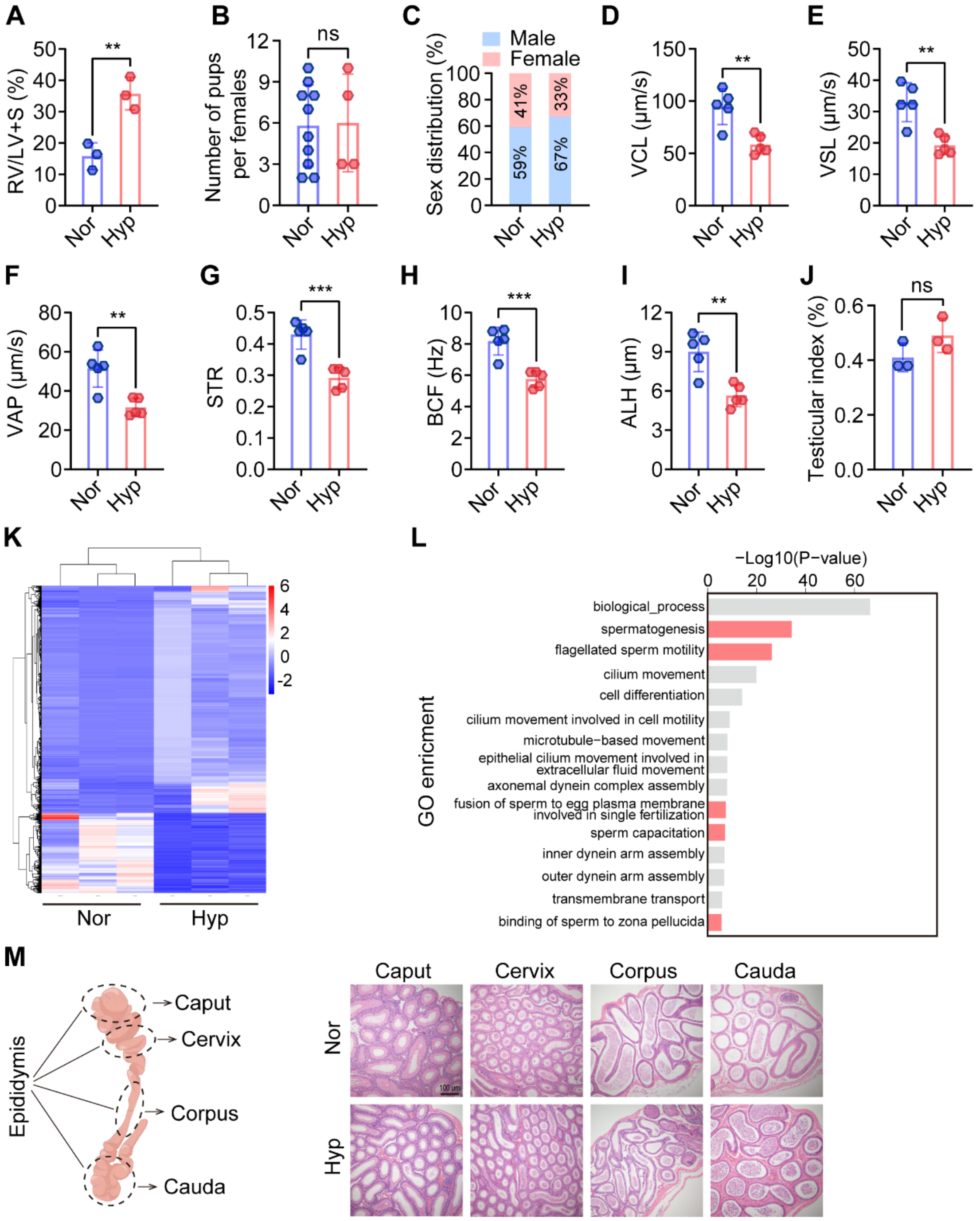

Supplementary Figure S1. Hypoxia damages sperm quality and epididymal function. (A) RVHI in Nor and Hyp mice. (B) Number of pups born to each female mated with Nor or Hyp male mouse (n=5 per group). (C) Gender distribution of offsprings. (D-I) Sperm VCL, VSL, VAP, STR, BCF, and ALH in Nor and Hyp mice. (J) Testicular index in Nor and Hyp mice, calculated as follows: testis weight/body weight *100% (n=3 mice per group). (K) Heatmap of the DEGs in the epididymis of Nor and Hyp mice. (L) Bar plot showing the top 15 pathways from the GO enrichment analysis of the DEGs. (M) Representative images of H&E staining of the epididymis from Nor and Hyp mice (n=3); scale bar=100 μm. The scale bar shown in the first panel applies to all panels in this figure. Data were analyzed using unpaired Student's t-test and presented as mean ± SD. *P<0.05, **P<0.01, ***P<0.001. RVHI: right ventricular hypertrophy index, calculated as: RVHI=[(RV

weight)/(LV+Septum Weight)] × 100 %; w: Weeks; Nor: normoxia; Hyp: hypoxia. VCL: curvilinear velocity; VSL: straight-line velocity; VAP: average path velocity; STR: straightness; BCF: beat cross frequency; ALH: amplitude of lateral head displacement; RV: right ventricle; LV: left ventricle; S: septum; DEGs: differentially expressed genes.

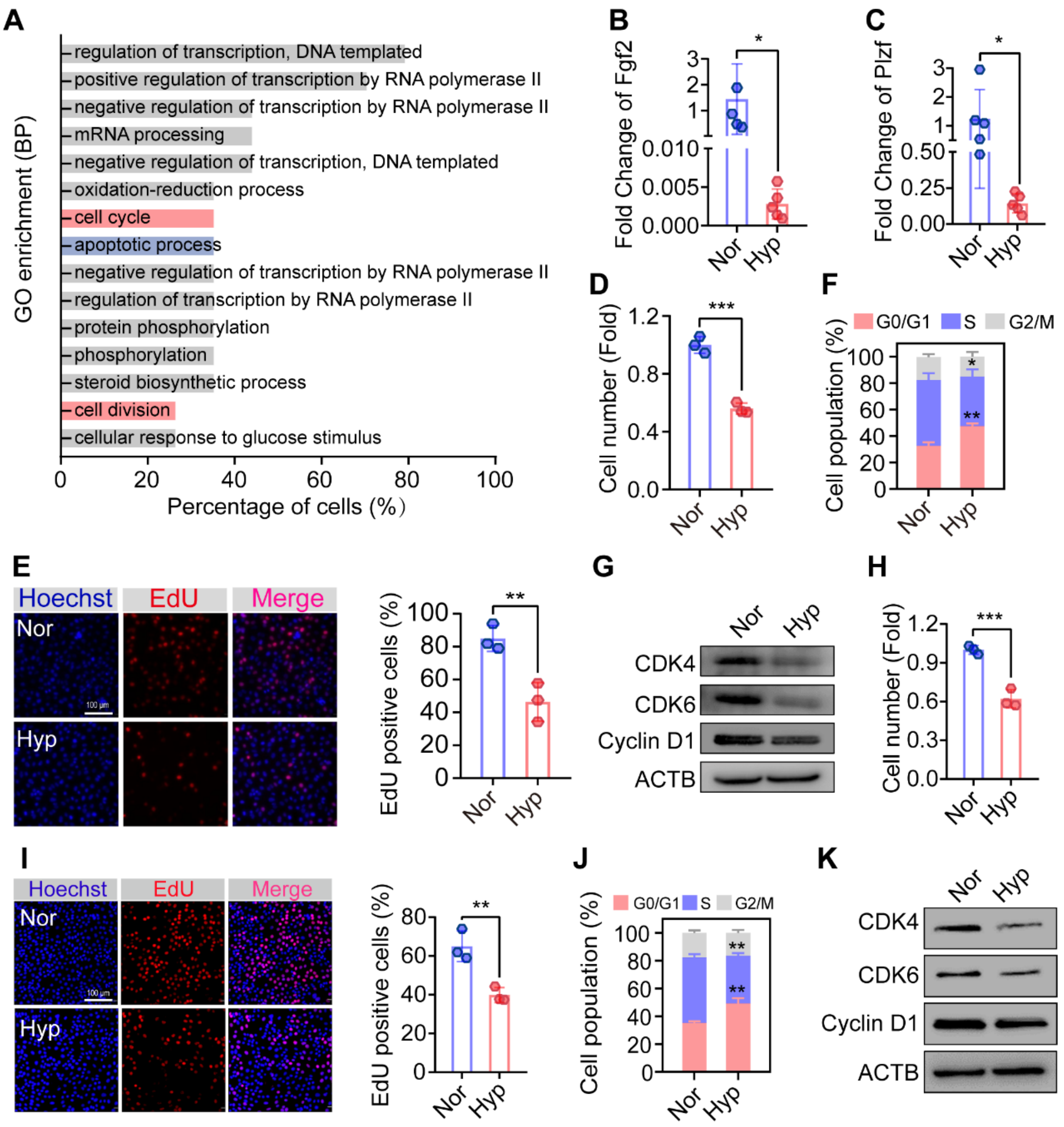

Supplementary Figure S2. Hypoxia impairs spermatogonia proliferation. (A) Bar plot of the top enriched GO biological process terms ranked by the percentage of genes in each term. (B-C) Relative expression of *Fgf2* and *Plzf* in the testes from Nor and Hyp mice (n=5 per group); scale bars=100 µm. (D) Relative cell number in GC-1 cells with Nor or Hyp exposure, as detected by CCK-8. (E) Representative image and relative cell number of EdU incorporation assay in GC-1 cells with Nor or Hyp exposure; scale bar=100 µm. (F) Cell cycle distribution in GC-1 cells with Nor or Hyp exposure, as detected by flow cytometry. (G) Protein levels of CDK4, CDK6 and cyclin D1 in GC-1 cells with Nor or Hyp exposure. (H-K) Relative cell number, representative image of EdU incorporation assay, cell cycle distribution, and protein levels of CDK4, CDK6 and cyclin D1 in GC-2 cells with Nor or Hyp exposure, as detected by CCK-8, EdU incorporation assay, flow cytometry, and western blotting; scale bar=100 µm. Data were analyzed using unpaired Student's t-test and presented as mean ± SD. ns, not significant (P>0.05), *P<0.05, **P<0.01, ***P<0.001. Nor: normoxia; Hyp: hypoxia.

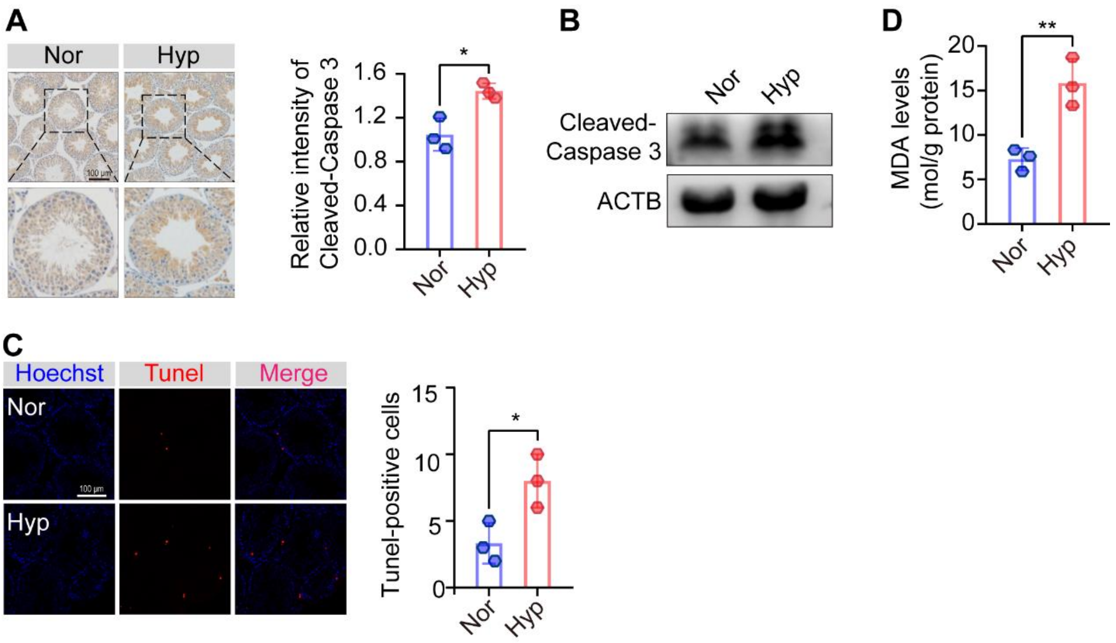

Supplementary Figure S3. Hypoxia promotes spermatogenic cells apoptosis in male mice. (A) Representative immunohistochemical images of cleaved Caspase 3 in testicular cross sections from Nor and Hyp mice (n=3 per group); scale bar=100 μm. (B) Protein expression of cleaved Caspase 3 in testes from Nor and Hyp mice (n=3 per group). (C) Representative image of TUNEL staining assay in testicular cross sections from Nor and Hyp mice (n=3 per group). (D) MDA levels in testes of Nor and Hyp mice (n=3 per group); scale bar=100 μm. Data were analyzed using unpaired Student's t-test and presented as mean ± SD. *P<0.05, **P<0.01. Nor: normoxia; Hyp: hypoxia. MDA: malonaldehyde.

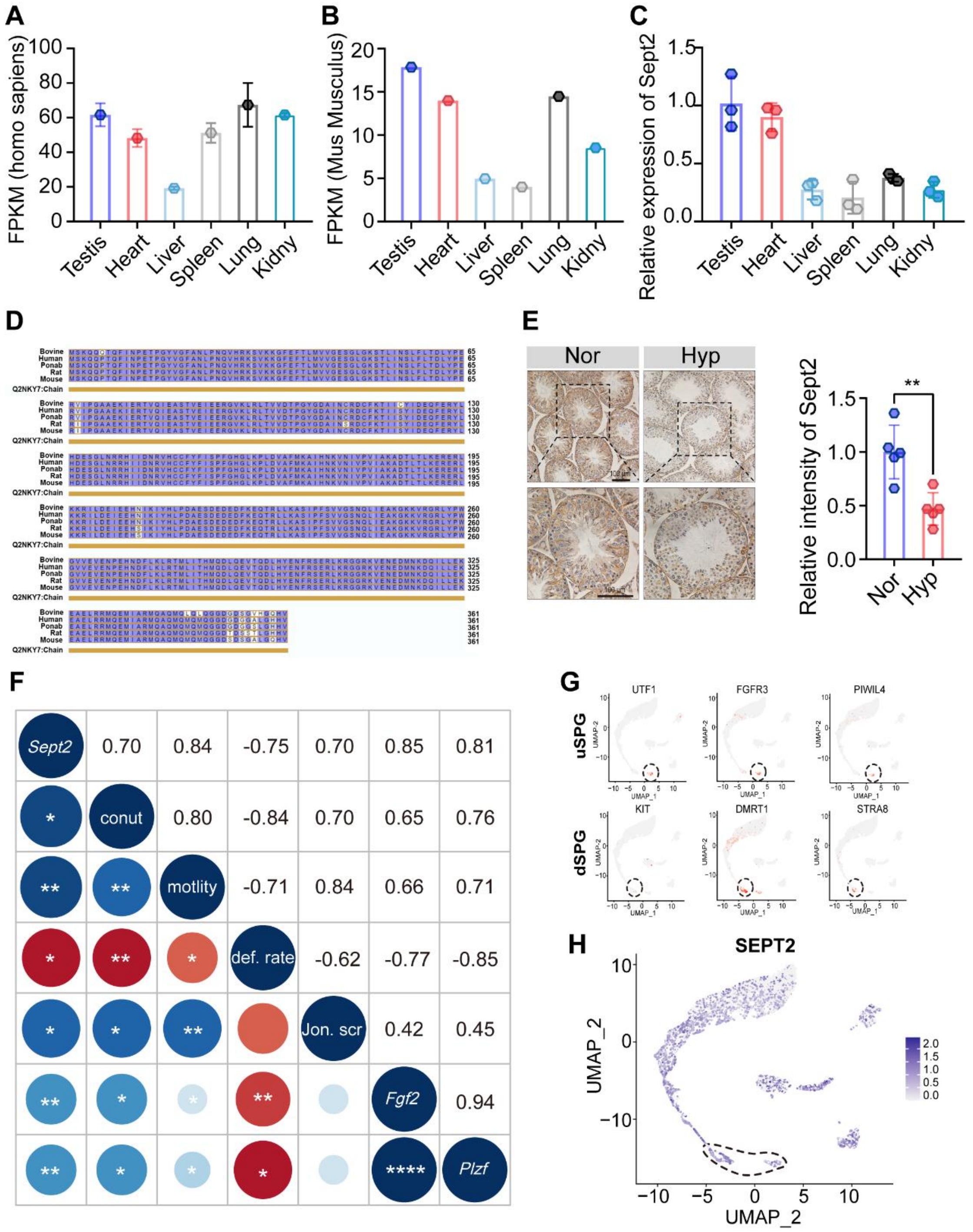

Supplementary Figure S4. Sept2 is reduced in hypoxic testes and is correlated with sperm quality and spermatogonial proliferation. (A-B) Expression levels of Sept2 mRNA in various organs of *Homo sapiens* (n=3-7) and *Mus musculus* (n=1) based on data from the NCBI database. (C) Relative mRNA expression of Sept2 in different organs from male mice (n=3). (D) Protein homology analysis of Sept2 in different mammals based on UniProt data. (E) Representative immunohistochemical images of Sept2 in spermatogonia from Nor and Hyp mice (n=5); scale bars=100 µm (F) Spearman correlation analysis of Sept2 mRNA level and reproductive phenotypes. The numerical values represent the R-values (G) Expression pattern of the markers for spermatogonia cluster projected on the UMAP plot: uSPG (*UTF1*, *FGFR3*, *PIWIL4*); dSPG (*KIT*,

DMRT1, *STRA8*). (H) UMAP plot showing the expression pattern of SEPT2 in spermatogonia. Data were analyzed using unpaired Student's t-test and presented as mean ± SD. **P<0.01. Nor: normoxia; Hyp: hypoxia; FPKM: fragments per kilobase of transcript per million mapped reads; UMAP: uniform manifold approximation and projection; uSPG: undifferentiated spermatogonia; dSPG: differentiating spermatogonia; Def. rate: deformity rate; Jon. Scr.: Johnson score.

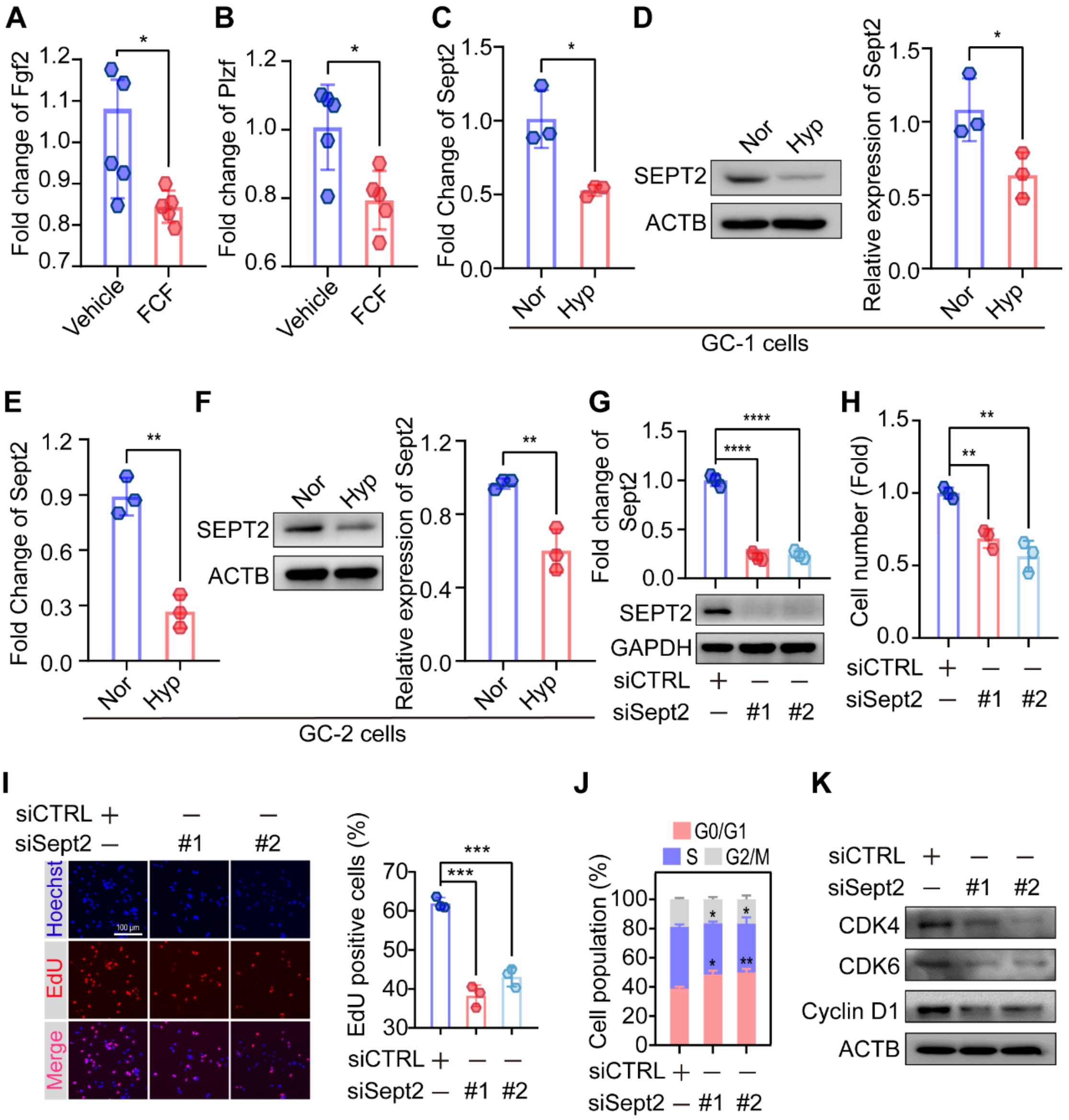

Supplementary Figure S5. Sept2 plays a crucial role in regulating spermatogonia proliferation. (A-B) Relative expression of *Fgf2* and *Plzf* in Vehicle and FCF mice (n=5 per group). (C-D) Relative mRNA and protein expression of Sept2 in Nor and Hyp GC-1 cells. (E-F) Relative mRNA and protein expression of Sept2 in Nor and Hyp GC-2 cells. (G) The efficiency of siRNAs targeting Sept2 in GC-2 cells. (H-J) Relative cell number, representative images of EdU incorporation assay, cell cycle distribution, and protein levels of CDK4, CDK6 and Cyclin D1 in GC-2 cells with Sept2 knockdown, as detected by CCK-8, EdU incorporation assay, flow cytometry, and western blotting; scale bar=100 µm. Data were analyzed using unpaired Student's t-test and presented as mean ± SD. *P<0.05, **P<0.01, ***P<0.001, ****P<0.0001. Nor: normoxia; Hyp: hypoxia; FCF: forchlorfenuron; siCTRL: negative control siRNA.

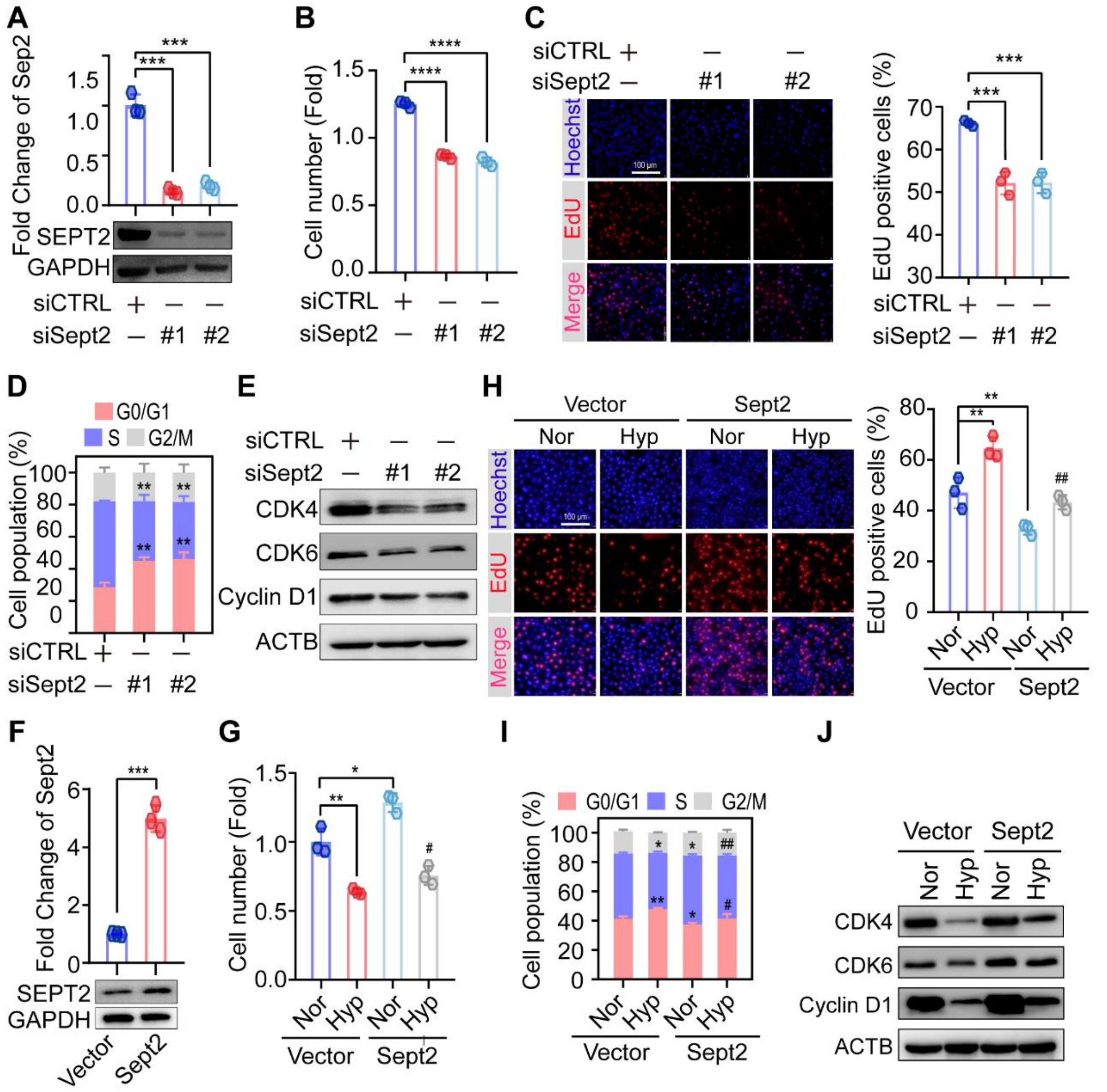

Supplementary Figure S6. Sept2 controls GC-1 cells proliferation. (A-E) Representative images of EdU incorporation assays, cell cycle distribution, and protein levels of CDK4, CDK6, and Cyclin D1 in GC-1 cells with Sept2 knockdown, as detected by CCK-8, EdU incorporation assays, flow cytometry, and western blotting; scale bar=100 μm. (F) Efficiency of Sept2 overexpression in GC-1 cells. (G-J) Relative cell number, representative EdU incorporation assay images, cell cycle distribution, and protein levels of CDK4, CDK6, and Cyclin D1 in Nor GC-1 cells with vector, Hyp GC-1 cells with vector, Nor GC-1 cells with Sept2 overexpression plasmid, and Hyp GC-1 cells with Sept2 overexpression plasmid, as detected by CCK-8, EdU incorporation assay, flow cytometry, and western blotting; scale bar=100 μm. Data were analyzed using unpaired Student's t-test and presented as mean ± SD. *P<0.05, **P<0.01, ***P<0.001, ****P<0.0001. ##, ###, The P value of Hyp with vector versus Hyp with Sept2 overexpression plasmid is less than 0.01, and 0.001. Nor: normoxia; Hyp: hypoxia; siCTRL: negative control siRNA.

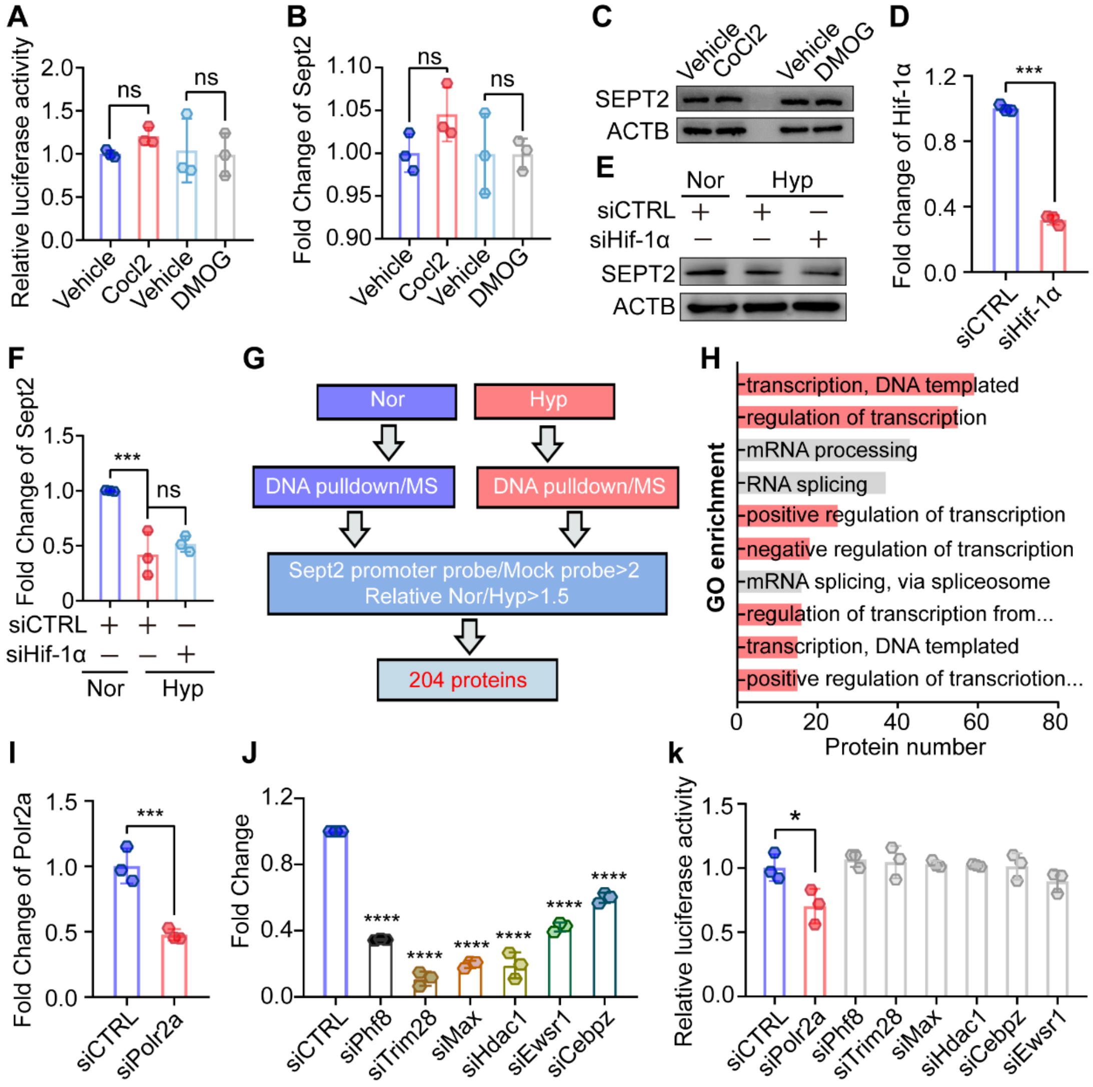

Supplementary Figure S7. Hypoxia inhibits Sept2 transcription independently of HIF-1α. (A) Dual-luciferase activity of the Sept2 promoter in GC-2 cells treated with $CoCl_2$ (200 μM) or DMOG (100 μM) for 24 h. (B-C) Relative mRNA and protein levels of Sept2 in GC-2 cells treated with $CoCl_2$ (200 μM) or DMOG (100 μM) for 24 h. (D) Efficiency of siRNA targeting Hif-1α. (E-F) Relative protein and mRNA levels of Sept2 in GC-2 cells treated with Nor, Hyp, or Hyp plus siHif-1α. (G) Schematic illustration of DNA pulldown/MS using the Sept2 promoter probe in GC-2 cells with or without hypoxia exposure. (H) GO enrichment analysis of proteins enriched by DNA pulldown/MS. (I) Efficiency of siRNA targeting Polr2a. (J) Efficiency of siRNAs targeting different TFs. (K) Transcription activity of Sept2 in GC-2 cells with different TFs knockdown. Data were analyzed using unpaired Student's t-test and presented as mean ± SD. ns, not significant ($P>0.05$), $^{*}P<0.05$, $^{***}P<0.001$, $^{****}P<0.0001$. Nor: normoxia; Hyp: hypoxia; TFs: transcription factors; POLR2A: RNA polymerase II subunit A; siCTRL: negative control siRNA; MS: mass spectrum.

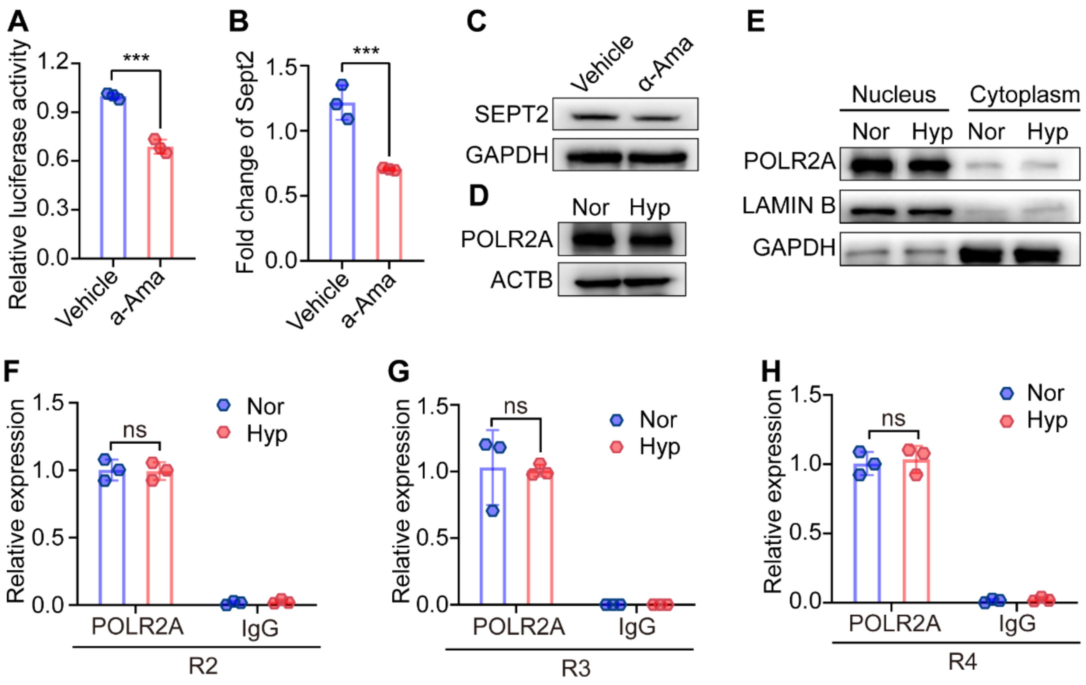

Supplementary Figure S8. POLR2A is involved in hypoxia-induced SEPT2 transcription deficiency. (A-C) Transcription activity, mRNA and Protein levels of Sept2 in GC-2 cells with vehicle or α-ama treatment. (D) Protein levels of POLR2A in Nor and Hyp GC-2 cells. (E) Nucleus and cytoplasm distribution of POLR2A in Nor and Hyp GC-2 cells. (F-H) ChIP-qPCR showing the relative expression of R2, R3, and R4 in Nor and Hyp GC-2 cells. Data were analyzed using unpaired Student's t-test and presented as mean ± SD. ns, not significant (P>0.05), ***P<0.001. Nor: normoxia; Hyp: hypoxia; POLR2A: RNA polymerase II subunit A; a-Ama: a-Amanitin. R2-R4: Region 2-Region 4.

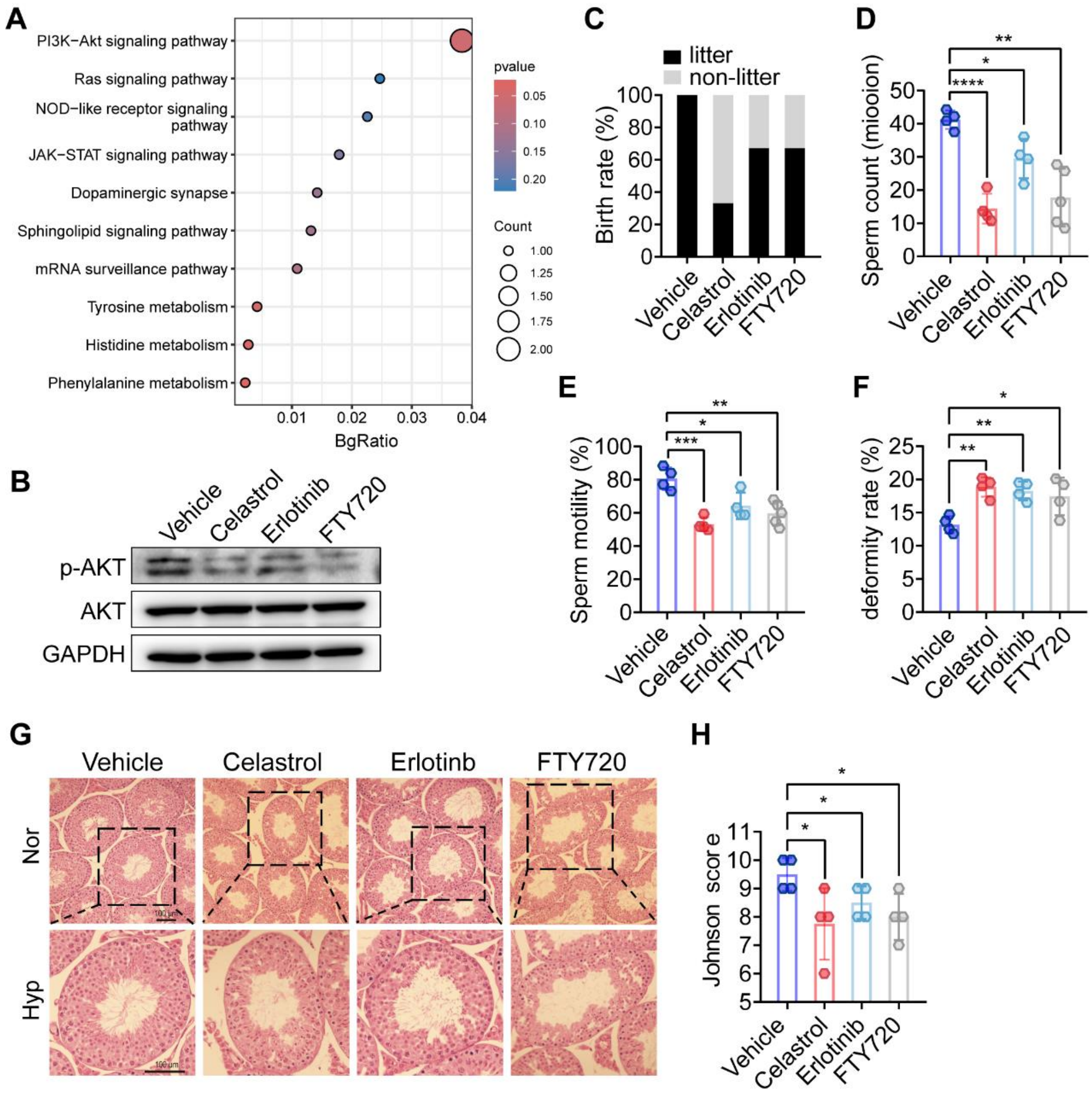

Supplementary Figure S9. Reproductive toxicities of PP2A agonists in male mice. (A) KEGG enrichment plot of the DEPs in GC-2 cells with or without Sept2 knockdown (B) Respective AKT and p-AKT levels in testes from males treated with different PP2A agonists (n=3 per group). (C) Male mice aged 6–8 weeks were treated with vehicle or different PP2A agonists. Fertility assessment showing the birth rate of female mice mated with Nor or Hyp males treated with different agonists (n=5 males and 10 females per group). (D-F) Sperm count and motility rate in mice treated with different PP2A agonists, as detected by CASA (n=4 per group). (F) Sperm deformity rate in mice treated with different PP2A agonists (n=4 per group). (G-H) Representative images of H&E staining and relative Johnson score in testicular cross sections from mice treated with different PP2A agonists (n=4 per group); scale bars=100 µm. Data were analyzed using unpaired Student's t-test and presented as mean ± SD. *P<0.05, **P<0.01, ***P<0.001, ****P<0.0001. PP2A: Protein phosphatase 2A; KEGG: kyoto encyclopedia of genes and genomes; DEPs: different expressed proteins; Nor: normoxia; Hyp: hypoxia; CASA: computer-assisted semen analysis.

Supplementary Table S1. Characteristics of the study participants

Characteristics	CTRL (n=19)	HA (n=24)	PH (n=6)	P value		
				CTRL *vs.* HA	CTRL *vs.* PH	HA *vs.* PH
Age (years)	26.05 ± 2.78	25.08 ± 2.54	27.83 ± 4.17	0.51	0.38	0.10
BMI (kg/m^2)	21.74 ± 2.21	22.99 ± 1.30	22.15 ± 1.57	0.06	0.54	0.87
Semen volume	4.05 ± 1.43	3.75 ± 1.85	3.33 ± 0.82	0.81	0.61	0.84
Liquefaction time	28.42 ± 10.28	36.04 ± 10.32	24.17 ± 6.65	0.04	0.64	0.03
Sperm concentration	60.37 ± 39.47	20.13 ± 8.70	38.00 ± 21.82	<0.0001	0.18	0.31
Sperm count	239.8 ± 160.5	62.05 ± 43.45	123.7 ± 70.65	<0.0001	0.08	0.48
PR (%)	45.74 ± 10.92	14.75 ± 9.35	22.67 ± 15.85	<0.0001	0.0001	0.26
PR+NP (%)	65.32 ± 14.11	43.88 ± 16.43	44.33 ± 20.27	0.0002	0.02	0.10
Head deformity (%)	89.71 ± 3.49	93.24 ± 3.23	91.08 ± 4.80	0.006	0.72	0.43
Tail deformity (%)	35.41 ± 10.44	46.13 ± 14.47	47.16 ± 14.70	0.029	0.18	0.99
Total deformity (%)	94.19 ± 1.31	95.77 ± 1.61	95.98 ± 1.93	0.005	0.06	0.96
Viability rate (%)	80.63 ± 6.07	65.74 ± 15.53	70.00 ± 12.67	0.0009	0.21	0.76
Smoking status	none	none	none	—	—	—
Drinking status	none	none	none	—	—	—
Testicular morphology	normal	normal	normal	—	—	—
Radiation exposure	none	none	none	—	—	—
Harmful substance exposure	none	none	none	—	—	—
Reproductive system infections	none	none	none	—	—	—
Family history of genetic disorders	none	none	none	—	—	—

Data represent the mean ± SD. Statistical analyses were performed by One-Way ANOVA. CTRL: normoxic healthy control; HA: high altitude hypoxia; PH: pathological hypoxia; BMI: body mass index. PR: progressive motility; NP: non-progressive motility; —: not applicable.

Supplementary Table S2. Details of Reagents and Kits

Reagents and Kits	Catalog Number	Supplier Name	Location
Sperm Morphology Staining Kit	G2571	Solarbio	Shanghai, China
PrimeScript™ RT Reagent Kit	RR047A	Takara	Shiga, Japan
TB Green Premix Ex Taq	RR420A	Takara	Shiga, Japan
BCA protein assay kit	P0009	Beyotime	Shanghai, China
iClick™ EdU Andy Fluor 594 Imaging Kit	A005	GeneCopoeia	Rockville, MD, USA
BD Pharmingen™ PI/RNase Staining Buffer	550825	BD Biosciences	San Jose, CA, USA
Dual-Luciferase Reporter Assay System	E1910	Promega	Madison, WI, USA
Nuclear and Cytoplasmic Protein Extraction Kit	P0028	Beyotime	Shanghai, China
PrimeStar® Max DNA Polymerase	R045A	Takara	Shiga, Japan
PureLink® Quick Gel Extraction Kit	K210025	Invitrogen	Carlsbad, CA, USA
Dynabeads™ Kilobase BINDER™ Kit	6010	Invitrogen	Carlsbad, CA, USA
Pierce Magnetic ChIP Kit	26156	Thermo Fisher Scientific	Waltham, MA, USA
Lipid Peroxidation MDA Assay Kit	S0131S	Beyotime	Shanghai, China
One Step TUNEL Apoptosis Assay Kit	C1089	Beyotime	Shanghai, China
HTF medium	MR-070-D	Merck Millipore	Billerica, MA, USA
Forchlorfenuron	68157-60-8	Aladdin	Shanghai, China
Okadaic acid	S1786	Beyotime	Shanghai, China
celastrol	HY-13067	MedChemExpress	Monmouth Junction, NJ, USA
erlotinib	HY-50896	MedChemExpress	Monmouth Junction, NJ, USA
FTY720	HY-11063	MedChemExpress	Monmouth Junction, NJ, USA
DMEM/HIGH GlUCOSE	SH30022.01	HyClone	Logan, UT, USA
Fetal Bovine Serum	A5256701	Gibco	Grand Island, NY, USA
Penicillin/gentamicin/streptomycin	C0223	Beyotime	Shanghai, China
$CoCl_2$	449776	Sigma-Aldrich	St. Louis, MO, USA
DMOG	S7483	Selleckchem	Houston, TX, USA
MG132	133407-82-6	Aladdin	Shanghai, China
cycloheximide	HY-12320	MedChemExpress	Monmouth Junction, NJ, USA
Lipofectamine 8000	C0533	Beyotime	Shanghai, China
RNAiso Plus	9109	Takara	Shiga, Japan
α-Amanitin	MED11734	Medbio	Shanghai, China
SC79	SF2730	Beyotime	Shanghai, China
CCK-8 reagent	HY-K0301	MedChemExpress	Monmouth Junction, NJ, USA

HRP detection reagents	WBKLS0050	Merck Millipore	Billerica, MA, USA
Protein A/G beads	P5048	Beyotime	Shanghai, China

Supplementary Table S3. Details of antibodies

Antibody Name	Catalog Number	Supplier Name	Location	Dilution Factor	Application(s)
Rabbit anti Septin2	ab179436	Abcam	Cambridge, MA, USA	1:1000<br>10 μl	WB<br>IP
Rabbit anti PCNA	60097-1	Proteintech	Wuhan, China	1:100	IHC
Rabbit anti KI67	28074-1	Proteintech	Wuhan, China	1:100	IHC
Rabbit anti CDK4	ab199728	Abcam	Cambridge, MA, USA	1:1000	WB
mouse anti CDK6	ab241554	Abcam	Cambridge, MA, USA	1:1000	WB
Rabbit anti Cyclin D1	ab16663	Abcam	Cambridge, MA, USA	1:1000	WB
Rabbit anti Caspase 3/ P17/P19	66470	Proteintech	Wuhan, China	1:500<br>1:100	IHC<br>WB
Rabbit anti MAGEA4	A11607	ABclonal	Wuhan, China	1:1000	WB
Rabbit anti DMRT1	28149-1	Proteintech	Wuhan, China	1:150	IF
Rabbit anti PLZF	A21185	ABclonal	Wuhan, China	1:200	IF
Rabbit anti POLR2A	A11181	ABclonal	Wuhan, China	1:1000<br>10 μl	WB<br>ChIP
Rabbit anti B56γ	A5480	ABclonal	Wuhan, China	1:1000<br>10	WB<br>IP
Rabbit anti IL7R	ab180521	Abcam	Cambridge, MA, USA	1:1000	WB
Rabbit anti BCL2L2	sc8392	Santa Cruz Biotechnology	Santa Cruz, CA, USA	1:1000	WB
Rabbit anti AKT	4691s	Cell Signaling Technology	Danvers, MA, USA	1:1000	WB
Rabbit anti pAKT	4060s	Cell Signaling Technology	Danvers, MA, USA	1:1000	WB
Rabbit anti Ubiquitin	A19686	ABclonal	Wuhan, China	1:1000	WB
Mouse Anti HA	AE008	ABclonal	Wuhan, China	1:2000<br>10 μl	WB<br>IP
Rabbit anti Lamin B	ab133741	Abcam	Cambridge, MA, USA	1:1000	WB
Mouse anti β-actin	66009-1	Proteintech	Wuhan, China	1:5000	WB
Mouse anti GAPDH	60004-1	Proteintech	Wuhan, China	1:5000	WB

Supplementary Table S4. List of the siRNAs

siRNA	Sense（5’-3’）	Antisense（5’-3’）
Sept2-1	UCAAUUUCAUCCAAAAUCCUU	GGAUUUUGGAUGAAAUUGAAG
Sept2-2	GAUGGUGGUUGGUGAAUCUTT	AGAUUCACCAACCACCAUCTT
Polr2a	ACAUAAGAACCAUCAAAGGAG	CCUUUGAUGGUUCUUAUGUCA
Phf8	AAAUUUUGAUCUUAUUAACAG	GUUAAUAAGAUCAAAAUUUCC
Trim28	AAUAACAAUCAGAUUGUAGUC	CUACAAUCUGAUUGUUAUUGA
Max	AUAUACUGGAUAUACUCUGUU	CAGAGUAUAUCCAGUAUAUGC
Hdac1	UCUGUUUUAACUCUUUUGGCU	CCAAAAGAGUUAAAACAGAGG
Ewsr1	UCUUGUUCAUCUUGACAACCC	GUUGUCAAGAUGAACAAGAGA
Cebpz	UUCUUUUGUCCUUGAAAUGAC	CAUUUCAAGGACAAAAGAAAA
Hif-1α	UGGAUAGCGAUAUGGUCAAUGTT	CAUUGACCAUAUCGCUAUCCATT

Supplementary Table S5. List of the primers

Gene	Forward（5'-3'）	Reverse（5'-3'）
Sept2	TCAAGGCCAGTATCCCATTC	GTTCTCCACCTCTACAACACCC
Glut1	GCAGTTCGGCTATAACACTGG	GCGGTGGTTCCATGTTTGATTG
Vegf	CTGCCGTCCGATTGAGACC	CCCCTCCTTGTACCACTGTC
Polr2a	GAGTCCAGAACGAGTGCATGA	ACAGGCAACACTGTGACAATC
Hif-1a	ACCTTCATCGGAAACTCCAAAG	CTGTTAGGCTGGGAAAAGTTAGG
Phf8	GGACTGGTTTCACGGCAGTT	GTAAGACCTCACAGTTAGGGCA
Trim28	CCCTGTCTACATTCGGCCTG	ACGATGTCTTTGGAGTAGCACT
Max	CAAGCGGGCTCACCATAATG	TGTTGCTTTGTCTAGGATTTGGG
Hdac1	TGAAGCCTCACCGAATCCG	GGGCGAATAGAACGCAGGA
Cebpz	TGGTACGACATGGAGTACAGC	GACTTCGTGCTCATACAGCTT
Ewsr1	TGTGCAGGGATATGGCACTG	ACCATCCTGTGGTCTGGTAGG
Fgf2	GCGACCCACACGTCAAACTA	TCCCTTGATAGACACAACTCCTC
Plzf	CTGGGACTTTGTGCGATGTG	CGGTGGAAGAGGATCTCAAACA
Magea4	ATGGAAAATCCCGATAACACCC	AGGACTTGGTATTCCACTACTGT
Actb	GTGCTATGTTGCTCTAGACTTCG	ATGCCACAGGATTCCATACC
Gapdh	GGTTGTCTCCTGCGACTTCA	TGGTCCAGGGTTTCTTACTCC
ChIP-P1	GTGGCACTCGGCTCTTTA	GAGGCAGACAGGTCAGAATA
ChIP-P2	CGATTGAAGGGAAGGACA	CACCATAGAGTGGTGCCTGT
ChIP-P3	ACCAGCACGTAGCCAATC	GGAGGAATAAGGTGTCCATT
ChIP-P4	CTTGGTAAAGGAGAAGAGGC	CTTGCCAACTCCTTCCTG
